# A Golgi and tonoplast localized *S*-acyl transferase is involved in cell expansion, cell division, vascular patterning and fertility in Arabidopsis

**DOI:** 10.1111/nph.12385

**Published:** 2013-06-25

**Authors:** Baoxiu Qi, James Doughty, Richard Hooley

**Affiliations:** 1 Department of Biology and Biochemistry, University of BathClaverton Down, Bath, BA2 7AY, UK; 2 State Key Laboratory of Crop Biology, Shandong Agricultural UniversityShandong, 271018, China

**Keywords:** Arabidopsis, DHHC-PAT, dwarfism, Golgi, protein *S*-acylation, tonoplast, trans-Golgi network, vascular patterning

## Abstract

- *S*-acylation of eukaryotic proteins is the reversible attachment of palmitic or stearic acid to cysteine residues, catalysed by protein *S*-acyl transferases that share an Asp-His-His-Cys (DHHC) motif. Previous evidence suggests that in Arabidopsis *S*-acylation is involved in the control of cell size, polarity and the growth of pollen tubes and root hairs.
- Using a combination of yeast genetics, biochemistry, cell biology and loss of function genetics the roles of a member of the protein *S*-acyl transferase PAT family, AtPAT10 (At3g51390), have been explored.
- In keeping with its role as a PAT, AtPAT10 auto-*S-*acylates, and partially complements the yeast *akr1* PAT mutant, and this requires Cys^192^ of the DHHC motif. In Arabidopsis AtPAT10 is localized in the Golgi stack, trans-Golgi network/early endosome and tonoplast. Loss-of-function mutants have a pleiotropic phenotype involving cell expansion and division, vascular patterning, and fertility that is rescued by wild-type AtPAT10 but not by catalytically inactive AtPAT10C^192^A. This supports the hypothesis that AtPAT10 is functionally independent of the other Arabidopsis PATs.
- Our findings demonstrate a growing importance of protein *S*-acylation in plants, and reveal a Golgi and tonoplast located *S*-acylation mechanism that affects a range of events during growth and development in Arabidopsis.

## Introduction

Many integral and peripheral membrane proteins involved in signal transduction or membrane trafficking can be subjected to covalent lipid modifications, such as myristoylation, farnesylation, prenylation and *S*-acylation. *S*-acylation (also known as *S*-palmitoylation) is the attachment of palmitic or stearic acid to cysteine residues via a labile thioester bond (Gleason *et al*., 2006; Resh, 2006a,b). It is widespread in eukaryotes, often coupled with myristoylation or prenylation, and increases the lipophilicity of the modified protein, thus enhancing its membrane association. Unlike other lipid modifications, it is reversible and can accommodate regulation by extracellular signals (Tsutsumi *et al*., 2008; Chini & Parenti, 2009). Cycles of de- and re-acylation of peripheral membrane proteins influence their membrane-association dynamics in both mammals and yeast. For example, Ras is subjected to a dynamic acylation pathway that mediates trafficking between Golgi and plasma membrane, and the correct membrane-localized functioning of α subunits of most heterotrimeric G proteins is dependent on *S*-acylation (Roth *et al*., 2006; Greaves & Chamberlain, 2007). Integral membrane proteins such as GPCRs, ion channels and SNARE proteins are *S*-acylated influencing fidelity of processing and transport to specific membranes and membrane microdomains, or altering conformation such that their activity or interaction with other proteins is modified (Resh, 2006a,b). *S*-acylation of cysteines in transmembrane domains (TMDs), can promote lateral diffusion into thicker microdomains rich in sphingolipid and cholesterol, or can tilt the TMD. Such changes shield or expose membrane-proximal amino acids that are targets for protein–protein interaction or post-translational modifications (Greaves & Chamberlain, 2007).

*S*-acylation is catalysed by protein *S*-acyl transferases (PATs) in yeast (Lobo *et al*., 2002; Roth *et al*., 2002) and in mammals (Fukata *et al*., 2004; Huang *et al*., 2004; Keller *et al*., 2004). PATs are integral membrane proteins with four to six TMDs and a cytoplasmic DHHC-containing Cysteine Rich Domain (DHHC-CRD) that is essential for catalytic activity (Montoro *et al*., 2011). *S*-acyl transferases are encoded by a seven member gene family in *Saccharomyces cerevisiae*, 15 predicted genes in *Caenorhabditis elegans*, at least 23 predicted genes in *Drosophila melanogaster*, mammals (Tsutsumi *et al*., 2008) and 24 in *Arabidopsis thaliana* (Batistič, 2012). In addition to the DHHC domain some *S*-acyl transferases also contain a PDZ-binding motif, others an SH3 domain, while other members contain multiple ankyrin repeats.

In plants, understanding of *S*-acylation is limited. A few proteins have been shown to be *S*-acylated and these are involved in Ca^2+^ signalling, movement of potassium ions, stress signalling and pathogenesis (Hemsley & Grierson, 2008; Hemsley, 2009). A proteomic approach identified > 500 potentially palmitoylated proteins in Arabidopsis (Hemsley *et al*., 2013); however, so far, only one plant *S*-acyl transferase has been characterized, Arabidopsis TIP1. The transcriptional null mutant alleles exhibit defects in cell size control, pollen tube, root hair growth and cell polarity (Hemsley *et al*., 2005). Recently, a survey of the genomics and localization of the 24 Arabidopsis PATs described the ubiquitous expression profiles of most PATs, and their complex targeting patterns in cellular membrane compartments which are different from their counterparts in yeast and mammals (Batistič, 2012).

As part of an effort to determine the biological functions of Arabidopsis PATs we analysed two T-DNA insertion lines (Alonso *et al*., 2003) of AtPAT10 (At3g51390), investigated the activity of the protein by expression in yeast and localized C-terminally YFP tagged AtPAT10 to the Golgi, trans-Golgi network (TGN) and tonoplast. Our results demonstrate that AtPAT10 is an *S*-acyl transferase involved in the regulation of cell expansion, cell division, vascular development, stature, shoot branching and fertility in Arabidopsis. This greatly expands the range of events in which *S*-acylation is involved in Arabidopsis and reveals a Golgi and tonoplast located *S*-acylation mechanism that affects a range of events during growth and development.

## Materials and Methods

### Plant materials and growth

Growth and transformation of Arabidopsis Col-0 were described previously (Qi *et al*., 2004; see also Supporting Information, Methods S1). SALK T-DNA lines, SALK_018436 and SALK_024964 were obtained from Nottingham Arabidopsis Stock Centre (NASC) and PCR-genotyped using primers, LBb1.3, zfLP and zfRP (Table S3), in two paired PCR reactions. Mutant lines were back crossed to Col-0, selfed for three generations and homozygotes isolated. The transcript levels in leaves of these two homozygotes as well as different tissues of the wild-type (WT) were detected by RT-PCR (see Methods S1).

### Cloning and mutagenesis

The coding region of *AtPAT10* was PCR-amplified with KOD polymerase (Merck Millipore) from a cDNA library in pUra-M (B. Qi & R. Hooley, unpublished). *AtPAT10C*^*192*^*A* was made by PCR mutagenesis (Qi *et al*., 2003) using primer pairs DHHCtoAF and ZFendnsE, and ZFbegK and DHHCtoAR (Table S3). PCR products were ligated in pJET1.2 (Fermentas) and sequenced. *AtPAT10* and *AtPAT10C*^*192*^*A* were cloned into pENTR/D and pENTR3C Dual (GATEWAY) to create entry clones pENTR-PAT10 and pENTR-PAT10C^192^A. These were cloned into pYES-DEST52 (GATEWAY) (with C-terminal V5 fusion) and pEarleyGate vectors (Earley *et al*., 2006) by GATEWAY recombination to generate yeast and plant expression vectors, respectively (see Methods S1).

### Complementation of yeast *akr1*

Wild-type BY4741 and *akr1* yeast were from EUROScarf. pYES-PAT10 and pYES-PAT10C^192^A were transformed into *akr1* (Hemsley *et al*., 2005). WT and *akr1* were transformed with pYES2 as positive and negative controls. For growth assays, yeast cells were grown in glucose minimal liquid media to stationary phase. A series of five- or 10-fold dilutions were made in sterile water from one OD600 of cells and 5 μl of each dilution was spotted onto two identical galactose minimal agar medium plates to induce protein expression. These were incubated at 25 and 37°C, respectively. Images were digitally scanned at 3 d. For microscopic observation, cells were grown in galactose minimal medium at 37°C to stationary phase and observed using DIC light microscopy with a ×100 objective lens. For DAPI staining, 1 OD600 of these cells were pelleted and resuspended in sterile water. DAPI (2.5 μg ml^−1^) from a 1 mg ml^−1^ stock solution was added and the cells were incubated at 25°C on a rotating mixer for 30 min before being observed under UV microscopy (see below).

### Auto-acylation of AtPAT10 by the acyl-biotinyl exchange (ABE) assay

Auto-acylation of AtPAT10 is detected by the *in vitro* ABE assay (Wan *et al*., 2007). pYES-PAT10-V5 and pYES-PAT10C^192^A-V5 were transformed into yeast strain YPH500 (Stratagene). Protein expression was confirmed by SDS-PAGE and Western blotting with anti-V5 antibody (Bethyl) and secondary alkaline phosphatase-conjugated antibody (Sigma). Two highly expressing clones from each construct were chosen for the ABE assay (Wan *et al*., 2007; see also Methods S1 for detailed procedures). AtPAT10/AtPAT10C^192^A captured on the beads was identified by Western blotting as above.

### Subcellular localization of AtPAT10

Leaf lower epidermis of mature plants, and primary roots of 5 d vertically grown *atpat10-1* seedlings, harbouring *35S:AtPAT10-YFP* (in pEarleyGate101) and *35S:AtPAT10-GFP* (in pEarleyGate103) were stained in FM4-64 (3.5 μM) in 0.5 × MS for 5 and 60 min, rinsed 3x in 0.5 x MS then imaged using a Nikon C1 LSCM (Nikon, Tokyo, Japan). GFP and YFP was visualized by excitation with a laser at 488 and 514 nm and emission was detected at 515/530 nm for both GFP and YFP, and 615 nm for FM4-64, using a 90i Eclipse microscope, with EZ-C1 software. Roots and hypocotyls were also imaged using an Olympus FV10i LSCM, excitation 473 nm, emission 480–580 nm in sequential mode.

For co-localization plants expressing AtPAT10-YFP were crossed with mCherry lines (Geldner *et al*., 2009) containing independent Golgi markers, Wave18R (Got1p), Wave22R (SYP32) and Wave127R (MEMB12), as well as Wave2R, 3R, 5R, 6R, 9R, 11R, 13R, 24R, 27R, 29R, 129R and 131R that mark other membrane compartments. F_1_ plants were selected on *Basta* and hygromycin (30 μg ml^−1^). Roots were visualized, with the same excitation/emission setting for YFP and excitation/emission at 559 nm/570–630 nm for mCherry using the 90i Eclipse microscope, with EZ-C1 software. YFP and RFP images were acquired by sequential line switching, allowing the separation of channels by both excitation and emission. Images were processed and merged using the IMAGEJ software (http://rsb.info.nih.gov/ij/).

### Light and scanning electron microscopy

Cross-sections of inflorescence stems were hand cut at the base, half way up, three quarters of the way up, and close to the tip. These were stained with Aniline Blue (0.05% in 0.67 M phosphate buffer, pH 8.0) and imaged under UV. For stem cell size measurements, a 3–5 mm piece of the base was fixed overnight in 50% ethanol, 5% acetic acid, 4% formaldehyde, dehydrated and embedded in resin (Technovit 7100 kit, Heraeus Kulzer, Germany). Sections (3–5 μm) were cut on a Leica microtome (LKB), stained in Toluidine blue (0.1% in 1% NaCl, pH 2.3) for 4 min and imaged using DIC, on a 90i Eclipse microscope (Nikon). For petal epidermal cell measurement, freshly opened flowers were fixed and cleared in 60% ethanol, 30% chloroform, 10% acetic acid for 24 h and imaged using the same microscope.

For scanning electron microscopy (SEM), tissues were fixed with 4% paraformaldehyde, and 5% glutaraldehyde, in 0.1 M CaC1_2_ and 0.1 M cacodylate buffer (pH 7.2) at 4°C for 16 h, rinsed with 0.1 M cacodylate buffer (pH 7.2), and post-fixed with a buffer containing 1% osmium tetroxide for 2 h at room temperature. Samples were then freeze-dried, coated with gold and observed by a JOEL scanning electron microscope (JSM-6480-LV).

## Results

### AtPAT10 has sequence similarity to, and predicted membrane topology characteristic of the PATs

*AtPAT10* (At3g51390) encodes a protein comprising 340 amino acids with a predicted molecular mass of 39.2 kDa. A BLASTP search against the Swissprot protein sequences at NCBI strongly suggests that AtPAT10 is a member of the zf-DHHC superfamily of *S*-acyl transferases. Although AtPAT10 has < 25% amino acid similarity to other functionally characterized PATs, it contains the conserved DHHC-CRD that is essential for *S*-acyl transferase activity (Fig. S1). TMD prediction algorithms, TMHMM v2.0, TMpred and SOSUI all predict four TMDs in AtPAT10 (Fig. S2 and data not shown). The DHHC-CRD, located between TMD2 and TMD3, together with both N- and C-termini, are predicted to be cytoplasmic (in Fig. S2, the cysteine C^192^ in the DHHC motif is highlighted in red). This topology is characteristic of all PATs identified so far (Hemsley *et al*., 2005; Fukata & Fukata, 2010; Batistič, 2012).

The Arabidopsis genome has at least 24 genes that encode potential PATs; alignment of these (Hemsley *et al*., 2005; Batistič, 2012) shows that all contain the DHHC-CRD and most also possess DPG and TTxE motifs of unknown function (SPG and STxE in AtPAT10) (Mitchell *et al*., 2006). AtPAT10 has 21.6–32.4% identity with these predicted proteins, with the greatest similarity in the DHHC-CRD region. It does not have N-terminal ankrin repeats.

### AtPAT10 is an *S*-acyl transferase

**Fig. 1:**
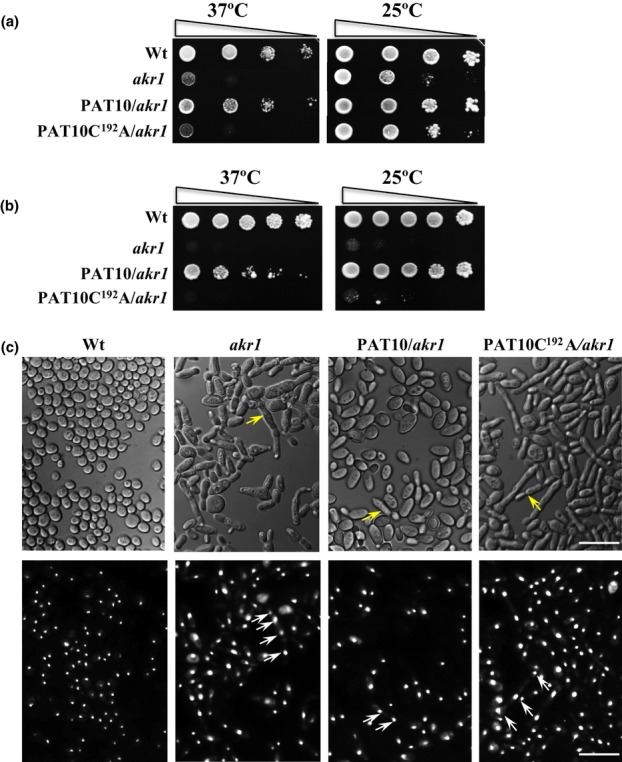
The Arabidopsis AtPAT10, but not AtPAT10C^192^A, can partially rescue growth defect of yeast temperature-sensitive *akr1* mutant that lacks a DHHC-PAT, AKR1. (a) Growth test. At the nonpermissive temperature of 37°C (left), wild-type (WT) yeast grew well but *akr1* did not. This growth defect was less obvious at 25°C because all genotypes grew well (right). Expressing AtPAT10 in *akr1* (PAT10/*akr1*) largely restored the growth inhibition by 37°C, but expressing AtPAT10C^192^A (PAT10C^192^A/*akr1*) did not. (b) Survival test. The cells of WT and AtPAT10 containing *akr1* (PAT10/*akr1*) continued to grow at both 37°C and 25°C after treatment at 37°C, but *akr1* and AtPAT10C^192^A (PAT10C^192^A/*akr1*) containing cells did not show much growth after this treatment. (c) DIC light (upper panel) and UV microscopy of DAPI (1 μg ml^−1^) stained cells (lower panel) of all four genotypes grown at 37°C. Arrows indicate multiple nuclei. Bars, 10 μm. Cells were transformed with empty vector pYES2 (WT and *akr1*), or with AtPAT10 and AtPAT10C^192^A (PAT10/*akr1*, PAT10C^192^A/*akr1*). Five microlitres of 5- (b) or 10-fold (a) serial dilutions from 1 OD600 cells were spotted on solid medium supplemented with 2% galactose and grown at 25°C or 37°C for 3 d.

Yeast AKR1p has been shown to be an *S*-acyl transferase (Roth *et al*., 2002). The loss-of-function mutant *akr1* shows various temperature-sensitive defects including elongated multinucleate cells and poor viability when grown at 37°C. At 25°C near normal growth occurs (Feng & Davis, 2000). To determine if AtPAT10 is an *S*-acyl transferase, *AtPAT10* and its point mutated variant *AtPAT10C*^*192*^*A* were expressed in *akr1* yeast cells. Figure 1(a) shows that at the nonpermissive temperature of 37°C, WT yeast grew well but *akr1* did not. This growth defect of *akr1* at 37°C was largely restored by expressing AtPAT10 because the transgenic *akr1* cells grew almost as well as the WT cells. However, expressing AtPAT10C^192^A in the *akr1* cells did not change the growth inhibition at this high temperature (Fig. 1a). The lack of growth exhibited by *akr1* and AtPAT10C^192^A in the *akr1* background at 37°C (Fig. 1a) was determined to be due to a severe reduction in viability as replating of these cells and incubation at both 25 and 37°C resulted in virtually no growth (Fig. 1b). Comparison of the cell morphology of all four genotypes grown at 37°C showed that while WT yeast cells were typically round, well dispersed and contain a single nucleus, the majority of *akr1* cells were elongated and clumped with multiple nuclei (arrows, Fig. 1c). The AtPAT10 expressing *akr1* cells looked more like WT than *akr1* mutant and the majority of them were well separated with a single nucleus (Fig. 1c). However, they were slightly elongated and a small number of cells had more than one nucleus (Fig. 1c, arrows). By contrast, cells from *AtPAT10C*^*192*^*A* transformed *akr1* were indistinguishable from *akr1* with many elongated cells possessing multiple nuclei (arrows, Fig. 1c). We confirmed by Western blotting that both AtPAT10 and AtPAT10C^192^A were expressed in *akr1* yeast (data not shown). Therefore, AtPAT10 partially rescues the phenotypes of *akr1* and this requires the Cys of the DHHC catalytic site.

**Fig. 2:**
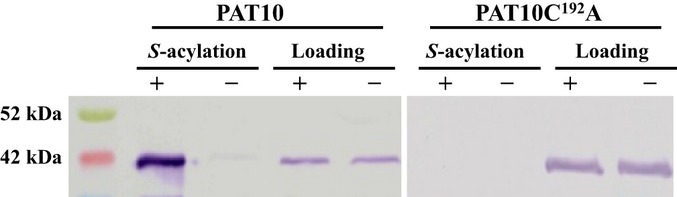
The Arabidopsis AtPAT10 is auto-acylated. AtPAT10 (PAT10) and AtPAT10C^192^A (PAT10C^192^A) were detected by Western blotting with anti-V5 antibodies by the alkaline phosphatase assay. The lanes in ‘*S*-acylation’ show the amount of AtPAT10 or AtPAT10C^192^A bound to the neutravidin beads with (+) or without (−) NH_2_OH treatment. ‘Loading’ controls showing equal amounts of protein were loaded (10% of the proteins used for ‘*S*-acylation’ assays). The molecular weight of AtPAT10 and AtPAT10C^192^A is *c*. 39 kDa. A strong band corresponding to AtPAT10 was detected in the +NH_2_OH treated sample in the ‘*S*-acylation’ lane, indicating that it is bound to an acyl group via a labile thioester linkage confirming that it is auto-acylated. However, no signal was detected for AtPAT10C^192^A and therefore it is not auto-acylated.

All *S*-acyl transferases characterized to date operate by a two-step process. First, the Cys residue of the DHHC motif is auto-acylated by binding an acyl group, such as palmitate. Following this, the acyl group is transferred to a Cys residue in the target protein (Hou *et al*., 2009; Mitchell *et al*., 2010; Jennings & Linder, 2012). This auto-acylation of the DHHC motif can be detected by the acyl-biotinyl exchange assay (Wan *et al*., 2007). To determine if AtPAT10 is auto-acylated at this Cys residue, yeast expressing AtPAT10 and AtPAT10C^192^A were subjected to ABE assay. For this the unmodified cysteine thiol groups on AtPAT10 and AtPAT10C^192^A in the yeast cell lysates were first blocked by the sulfhydryl reactive reagent N-ethylmaleimide (NEM). They were then treated with the *S*-acyl group cleavage reagent hydroxylamine (+NH_2_OH) to release thioester-linked palmitoyl moieties, restoring the modified cysteine to thiols (-SH) which were then biotinylated using a thiol-reactive biotinylation reagent biotin-HPDP. The biotinylated proteins can be immobilized onto neutravidin agarose beads and detected by Western blotting. In the negative control (-) NH_2_OH was omitted so that free sulfhydryls were not generated; therefore, proteins do not undergo biotinylation and hence are not detected. Figure 2 shows that when NH_2_OH was present AtPAT10 was biotinylated and detected by Western blotting, indicating that it is bound to an acyl group via a labile thioester linkage confirming that it is auto-acylated. However, no signal was detected for AtPAT10C^192^A; therefore, the mutant is not biotinylated and hence not auto-*S*-acylated. Our data clearly demonstrate that AtPAT10 is auto-acylated and this lipid modification requires the Cys residue in the DHHC motif. Taken together these observations strongly suggest that AtPAT10 is an *S*-acyltransferase.

### AtPAT10 is expressed in seedlings and roots, shoots, leaves and flowers of mature plants

In order to understand when and where the gene is expressed RT-PCR was carried out to amplify the full length cDNA of *AtPAT10* from seedlings and various tissues of mature plants. This showed that *AtPAT10* transcripts can be detected in seedlings and in roots, shoots, leaves and flowers of mature plants with the strongest signals detected in roots and floral organs (Fig. S3). This is consistent with publicly available microarray data (TAIR) (Batistič, 2012), suggesting that *AtPAT10* is expressed ubiquitously. It may therefore be involved in a broad range of events during Arabidopsis growth and development.

### AtPAT10 T-DNA mutants are semi-dwarfed with greatly reduced seed production and greater longevity

**Fig. 3:**
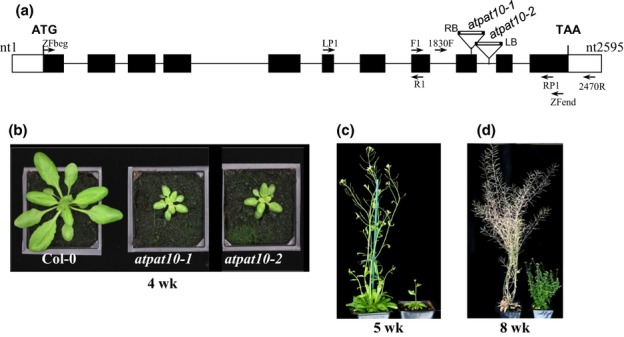
Characterization and complementation of 2 SALK T-DNA mutant lines of *AtPAT10* from Arabidopsis. (a) Schematic structure of the *AtPAT10* gene and positions of 2 T-DNA insertions, *atpat10-1* (SALK_018436), *atpat10-2* (SALK_024964). Positions of relevant primers are indicated. Solid boxes represent exons, empty boxes untranslated regions and lines introns. (b) 4-wk-old WT Col-0 (left) and *atpat10-1* (middle) and *atpat10-2* (right) plants; (c) 5-wk-old *atpat10-1* (right) and WT Col-0 (left) plants; (d) 8-wk-old *atpat10-1* (right) and WT Col-0 (left) plant.

In order to determine the function of AtPAT10 in Arabidopsis, two independent T-DNA insertion lines in *AtPAT10* (SALK_018436 and SALK_024964) were obtained from the Nottingham Arabidopsis Stock Centre (NASC) (Alonso *et al*., 2003). Plants homozygous for both insertion events were identified. The exact insertion sites were determined by sequencing the junction regions between the T-DNA and *AtPAT10* that show that *atpat10-1*, isolated from SALK_018436, has the T-DNA inserted between positions nt1948 and 1955 in exon 9, resulting in a 7-bp deletion (Fig. 3a). The second line, *atpat10-*2, was isolated from SALK_024964 which has a T-DNA insertion located in intron 9 at nt2047 (Fig. 3a). RT-PCR on mRNA isolated from leaves of Col-0 and both mutant lines show that no full-length transcripts, or transcripts across the T-DNA insertion sites, were detected from either T-DNA insertion mutant. However, truncated transcripts were detected upstream of the T-DNA insertions (Figs 3a, S4).

Detailed phenotypic analysis revealed that both *atpat10-1* and *atpat10-2* were semi-dwarf, had greatly reduced fertility, over-proliferation of shoot branching, reduced senescence, and continued to grow under long days until they were at least 4 months old (Fig. 3b and data not shown). Germination and establishment of the mutant seedlings on both soil and phytogel medium was poor compared with the WT (Fig. S5). Both *atpat10-1* and *atpat10-2* were found to exhibit identical phenotypes (data not shown).

The semi-dwarf phenotype of the mutants was apparent in young seedlings at the first leaf stage. At the rosette stage (4-wk-old plants), although the mutant and WT had produced the same number of leaves, the area of the first fully expanded leaf in the mutant was only 14.2% of the WT (Fig. 3b, Table S1). The length and width of the leaf as well as the length of the petiole were reduced in the mutant (Table S1). The phenotypic defects in the mutant became more pronounced after the plant started to flower (Fig. 3b). At 5 wk, when the WT had produced one primary and four secondary inflorescences, the mutant had developed a primary inflorescence that was only 25% of the height of WT (Fig. 3b, Table S1). This reduction in height was due to the reduced length of the internodes (Table S1) suggesting that the mutation might affect cell size and/or number. When WT plants had shed their seed and senesced (8 wk) (Fig. 3b), mutants were still green and produced additional secondary and lateral inflorescences from the axils of both rosette leaves and cauline leaves, resulting in a ‘bushy’ phenotype with > 50 inflorescence branches in the mutant plant after *c*. 12 wk (data not shown) compared to 14 in mature WT (Fig. 3b).

### Reduced seed set is caused by a combination of defects in the flower

**Fig. 4:**
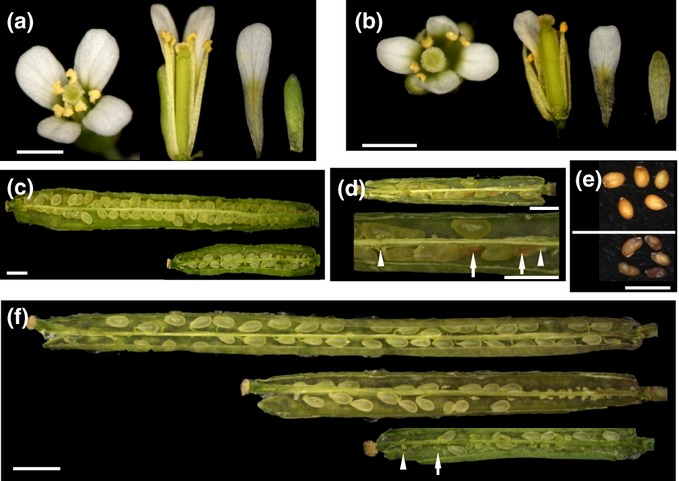
Comparison of the reproductive organs and seed development from WT Col-0 and the AtPAT10 mutant line *atpat10-1* from Arabidopsis. (a) Col-0 flower. (b) *atpat10-1* flower. (c) Col-0 (upper) and *atpat10-1* (lower) siliques at 5 d after pollination (DAP). (d) Seed abortion at 7 DAP in mutant. Arrows indicate aborted seeds and arrowheads unfertilized ovules. (e) Mature seeds from Col-0 (top) and *atpat10-1* (bottom). (f) Reciprocal crossing between Col-0 and *atpat101-1*. All siliques were at 5 DAP. Top, WT pollinated by WT (WT/WT); middle, WT pollinated by mutant (WT/*atpat10-1*); bottom, mutant pollinated by WT (*atpat10-1*/WT), arrow indicates smaller yet fertilized seeds, arrowhead indicates an unfertilized and shrivelled ovule. Note variations in seed size in *atdpat10-1*/WT silique. Bar, 1 mm.

The mutant plants produced small siliques, the vast majority of which contained no seed. A small number of siliques were found with seeds but typically these contained no more than 5 seeds compared to > 50 seeds per silique in the WT plants. Seeds of the homozygous mutant were smaller than WT, more irregular in shape and more pigmented (Fig. 4e, bottom). The mutant flowers are smaller than the WT and their stamens are proportionally shorter than the carpel and were rarely seen growing above the stigma (Fig. 4b). The anthers appear smaller, darker and although they dehisce they release less pollen than the WT. In fully open flowers very few pollen grains were seen on the stigma compared with the WT this is most likely due to the stamens not coming into contact with the stigma as well as the poor release of pollen.

We investigated the poor seed set in the mutant and found that the number of ovules per pistil was 40 compared to 60 in the WT (Table S1). At 5 d after pollination (DAP), the mutant siliques had 15 ± 3 seeds indicating that less than half of the ovules had been pollinated (Fig. 4c, bottom). However, some seeds started to shrivel at 7 DAP (arrows in Fig. 4d) and typically only *c*. 5 seeds remained in mature siliques (Fig. 4e, bottom).

In order to determine whether the pollen and/or ovules contribute to the lower fertility observed in these mutants, manual self-pollination and reciprocal crosses were performed. Mutant plants that were manually self-pollinated produced seed in every silique. This demonstrates that the failure of the pollen to come into contact with the stigma because of the short stamens is a major determining factor in the reduced fertility. However, despite the larger amount of pollen deposited on the stigma, seed set was still only comparable to that of naturally self-pollinated mutant flowers with typically no more than 5 seeds in the mature silique. Cross-pollination using WT pollen on mutant stigma again only resulted in *c*. 5 seeds per mature silique (Fig. 4f) suggesting that there is also a defect in the female organs. To assess the fertility of pollen from the mutant we cross-pollinated WT stigmas using mutant pollen. This resulted in fewer seeds being set compared with WT pollen and those that were fertilized were found only in the top half of the ovules (Fig. 4f, bottom). This suggests that the pollen tubes of the mutant were partially defective in growth. Nevertheless, all the ovules fertilized by the mutant pollen developed to maturity (Fig. 4f, top). These results indicate that the reduced fertility of the mutant is caused by a combination of factors including short stamens, reduced production and release of pollen, reduced number of ovules, defective pollen tube growth, and abortion of fertilized ovules in the mutant.

### Semi-dwarf phenotype is caused by a reduction in cell expansion and division

**Fig. 5:**
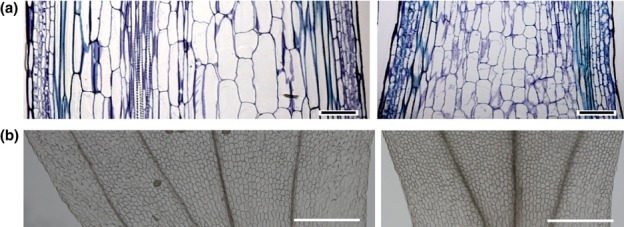
*atpat10* mutant Arabidopsis has smaller and fewer cells. (a) Longitudinal sections (resin embedded) of the base of the WT Col-0 (left) and *atpat10-1* (right) primary inflorescence stems stained with Toluidine blue. Sections were taken from the widest part of the stems. (b) Epidermal cells immediately above the elongated cells of the claw of the adaxial side of cleared petals from fully opened flowers of Col-0 (left) and *atpat10-1* (right). Thirty day-old plants were used. Bars, 100 μm.

In order to establish the basis of the semi-dwarf nature of the mutant, we measured cell size and number in primary inflorescence stems and petals of fully open flowers of WT and *atpat10-1* plants. The longitudinal section area of parenchyma cells in the pith of the stem of the mutant was *c*. 60% of that of the WT cells (Figs 5a, S6, Table S2). Similar results were obtained with epidermal cells of petals (Fig. 5b, Table S2). The number of epidermal cells in petals of the mutant in the widest part of the blade was some 32% less than that of the WT. Similarly, the number of parenchyma cells in the central region of the pith of the mutant stems was *c*. 57% less than the WT (Table S2, Fig. S6). Therefore, the semi-dwarf nature of the mutant appears to be the result of both a reduction in cell number and cell size in these tissues. These observations strongly suggest that the loss of PAT activity in *atpat10* affects the control of cell division and cell expansion.

### Loss of *AtPAT10* function affects vascular development

**Fig. 6:**
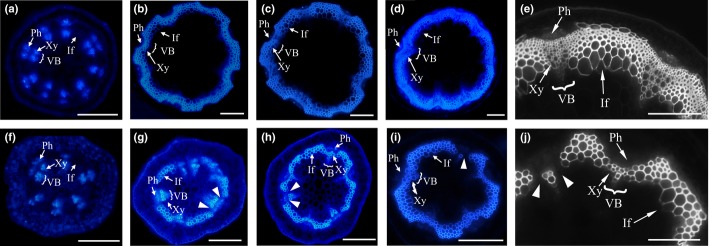
Vascular patterning in the stem of *atpat10-1* mutant Arabidopsis is altered. UV microscopy images of hand-cut sections after staining with Aniline Blue from various positions along the inflorescence stems. (a–e) WT Col-0. (f–j) *atpat10-1*. (a, f) Sections taken just below the apical meristem; (b, g) three-quarters of the way up; (c, h) halfway up; (d, e, i, j) base of the stem. Discontinuities between the lignified interfascicular fibres (If) and xylem cells (Xy) in the vascular bundles (VB) can be seen in the *atpat10-1* sections from three quarters to the base of the inflorescence stem (arrowheads in g–j). Ph, phloem. Bars: (a–d, f–i) 200 μm; (e, j) 100 μm.

We investigated the short, thin inflorescence stem of *atpat10* and found that its primary inflorescence stem had a significant defect in development of vascular bundles and interfascicular tissue compared to the WT (Figs 6, S6).

**Fig. 7:**
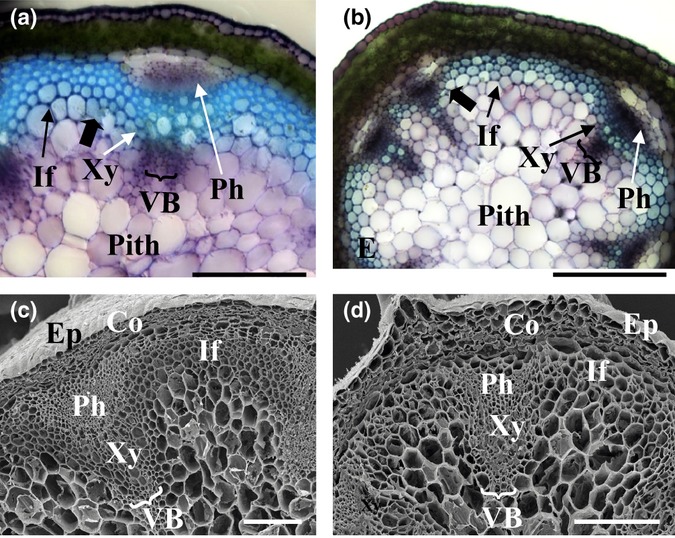
Light and scanning electron microscopy images from the base of inflorescence stems of 30-d-old Arabidopsis plants. (a, c) WT. (b, d) *atpat10-1*. Thick black arrows in (a) and (b) indicate the junction areas between the vascular bundles and the interfascicular fibre. If, interfascicular fibre; VB, vascular bundles; Ph, phloem; Xy, xylem, Co, cortex; Ep, epidermis. Bars, 200 μm.

At the base of the stem in WT, eight vascular bundles were distributed in an ordered radial pattern separated by interfascicular tissue of differentiated fibres. In the same region of the *atpat10-1* stem there were only five to six bundles that were smaller with fewer cells in both phloem and xylem (Fig. S6). Under UV Aniline Blue-stained mutant tissue revealed an absence of lignified cells at the junctions between xylem and interfascicular tissue, resulting in discontinuity of the lignified ring of cells (arrowheads in Fig. 6i,j). There are also fewer lignified cells at the outer periphery, and in the bundles. Higher power images of Toluidine blue-stained sections show that the lignified cells (stained blue) at the junction areas between the vascular bundles and the interfascicular fibre are less obvious, or absent (compare thick black arrows in Fig. 7a,b). Scanning electron microscopy (SEM) confirmed that the vascular bundles and interfascicular fibre are defective in the mutant (compare Fig. 7c and d).

Half way up the WT stem, *c*. 4 layers of interfascicular fibre cells were lignified as intensely as xylem bundles (Fig. 6c). In the mutant, however, there were only *c*. 2 layers and there were fewer lignified cells in the xylem bundles. The discontinuity of the ring of lignified cells was more pronounced than in the base (Fig. 6h, arrowheads).

Three-quarters of the way up the stem there were 3–4 cell layers of lignified interfascicular fibres between vascular bundles forming an arch-shaped pattern between bundles in the WT (Fig. 6b). By contrast, the interfascicular region in the *atpat10-1* stem had only 1 or 2 layers of liginified cells and because cells between xylem bundles and the interfascicular regions were rarely seen to be lignified, this caused discontinuity in this region (Fig. 6g, arrowheads). Immediately below the apex there were fewer vascular bundles in *atpat10-1* (6) compared to the WT (9) although they are similar in organization. Interfascicular cells were not visible by Aniline Blue (Fig. 6a,f), or Toluidine blue staining (data not shown), indicating that no fibre cells were differentiated in this part of the stem.

### The *atpat10* phenotypes are caused by the loss of AtPAT10 PAT activity

**Fig. 8:**
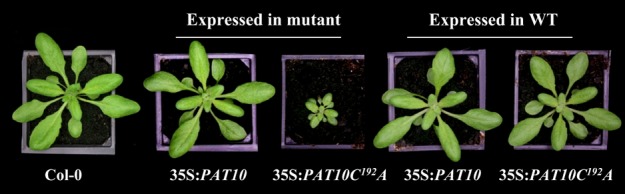
Complementation of 4-wk-old *atpat10* Arabidopsis mutant and ectopic overexpression. *35S:AtPAT10* complements the phenotype of *atpat10* but 35S:*AtPAT10C*^*192*^*A* does not. Expressing these constructs in the WT Col-0 did not affect the phenotype of 4-wk-old plants.

In order to determine if phenotypes of both mutants are caused by the loss of AtPAT10 PAT activity, we first complemented *atpat10-1* and *atpat10-2* plants by transforming them with constructs expressing AtPAT10 without any tags, an N-terminal FLAG-tagged AtPAT10 fusion protein, and C-terminal YFP- and GFP-tagged AtPAT10 fusion proteins, all under the control of the CaMV 35S promoter. Because homozygous mutant plants produce very few fertile flowers heterozygous plants were transformed. The transformants in the homozygous *atpat10-1* or *atpat10-2* backgrounds were isolated by PCR-based genotyping (Methods S1). All *35S:AtPAT10*, *35S:FLAG-AtPAT10*, *35S:AtPAT10-GFP* and *35S:AtPAT10-YFP* lines exhibited phenotypes that were indistinguishable from WT plants (Fig. 8, data not shown). Therefore, AtPAT10 and the three fusion proteins can rescue the phenotypes of *atpat10-1* and *atpat10-2*. This demonstrates that the phenotype of the mutants described above is caused by loss of function of *AtPAT10*.

We next tested whether or not the phenotype of *atpat10* was caused by loss of AtPAT10 PAT enzyme activity by transforming heterozygous *atpat10-1* plants with a construct expressing a C-terminal YFP tagged AtPAT10C^192^A mutant. Eight independent transgenic lines in the homozygous *atpat10-1* background were isolated. These transformants exhibited phenotypes identical to *atpat10-1* (Fig. 8). The presence of the *35S:AtPAT10C*^*192*^*A* construct, and the Cys to Ala point mutation in these transgenics, were confirmed by RT-PCR and sequencing the product amplified from total RNA isolated from leaf tissue. The transcript level of *AtPAT10C*^*192*^*A* was comparable to that of *AtPAT10* in the WT (data not shown). Expression of the C-terminal YFP tagged AtPAT10C^192^A protein was confirmed by LSCM (data not shown). This demonstrates that the phenotype of the mutants is caused by loss of PAT activity of AtPAT10, and that AtPAT10 is functionally independent of the other 23 Arabidopsis PATs.

Ectopic overexpression of AtPAT10 and AtPAT10C^192^A did not alter the phenotype of WT plants in terms of stature, flowering time, number of flowers and seed-set of all the transgenic plants cultivated (Fig. 8). This is not inconsistent with AtPAT10 being expressed ubiquitously.

### AtPAT10 is localized in the Golgi stack, trans-Golgi network and tonoplast

**Fig. 9:**
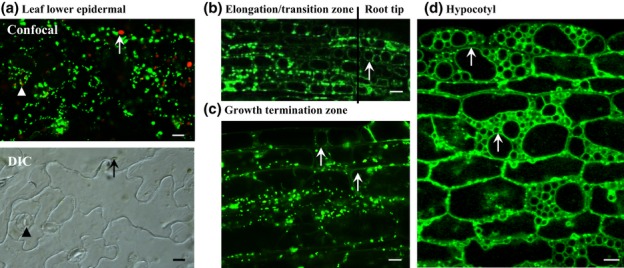
Subcellular localization of Arabidopsis AtPAT10 observed by LSCM. (a) In leaf epidermal cells. AtPAT10-GFP occurs as green punctuate structures dispersed along the plasma membrane (PM). Arrows indicate chloroplast shown (red in confocal image), arrowheads, guard cells. (b) In the primary root tip and ‘elongation/transition zone’ of 5-d-old seedling. AtPAT10-YFP is localized in the tonoplast (arrows) as well as in punctuate structures in cells of the elongation/transition zone. (c) In cells of the ‘growth terminating zone’ of primary root of the same age seedlings as in (b). AtPAT10-YFP is shown as punctuate structures along PM as well as in the tonoplast (arrows). (d) In hypocotyl. AtPAT10-YFP is located in the tonoplast (arrows) as well as punctate structures. Bars, 10 μm.

**Fig. 10:**
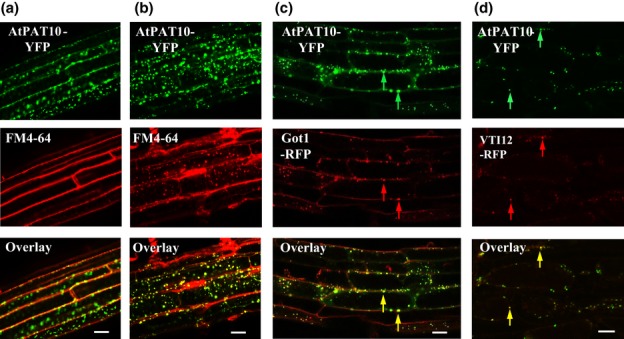
Subcellular localization of Arabidopsis AtPAT10 in the primary root. (a, b) Images were taken after 5 (a) and 60 min (b) of FM4-64 staining. After 60 min (b) AtPAT10-YFP (green) largely co-localizes with FM4-64 (red) in discrete punctae. (c) Some AtPAT10-YFP (green) co-localizes with the Golgi stack marker Got1 (red). Yellow arrows indicate co-localization between AtPAT10-YFP (green arrows) and Got1 (red arrows). (d) Some AtPAT10-YFP (green) co-localizes with the TGN/early endosome marker VTI12 (red). Yellow arrows indicates co-localization between AtPAT10-YFP (green arrows) and VTI12 (red arrows). Bars, 20 μm.

AtPAT10 is predicted to have 4 TMDs, therefore, it is likely to be localized in a membrane. To determine its subcellular localization, we observed by LSCM one of the transgenic lines rescued with *35S:AtPAT10-GFP*. The results are shown in Fig. 9 and video clips (Movies S1–S4). In leaf epidermal cells, AtPAT10-GFP occurs as small discrete punctae that might be a component(s) of the endomembrane system (Fig. 9a). In the root and hypocotyl of 5-d-old seedlings we observed AtPAT10-GFP in similar small discrete punctae, and in pro-vacuole and vacuole membranes (Fig. 9b–d). In many cells of the root tip region, and in cells close to the apex of the hypocotyl, we observed AtPAT10-GFP in the membrane surrounding large numbers of pro-vacuoles and in the associated central vacuole (Fig. 9b,d). Time-lapse imaging showed some of these pro-vacuoles coalescing with the growing central vacuole (Movie S1). Primary root cells of the elongation and growth terminating zones (Verbelen *et al*., 2006) contained a single large central vacuole that accounts for most of the cell volume (Fig. 9c). In these the AtPAT10-GFP fluorescence was less obviously associated with the vacuolar membrane but mainly as small discreet punctae dispersed in the cells. To more precisely determine in which membrane compartment(s) AtPAT10-YFP was localized, we first stained live primary roots of the 35S:AtPAT10-YFP transgenic line with the fluorescent styryl dye FM4-64. FM4-64 is a membrane-selective dye that fluoresces significantly only when it is in a lipid-rich environment. In plant cells it is rapidly incorporated into the PM followed by time-dependent appearance in small discrete intracellular organelles that might be components of the endocytotic pathway, before reaching the vacuole membrane (Ueda *et al*., 2001; Bolte *et al*., 2004). CLSM analysis revealed that after 5 min staining with FM4-64, the PM was clearly labelled in cells of the growth-terminating zone but there was no co-localization with AtPAT10-GFP (Fig. 10a); therefore, AtPAT10-GFP does not appear to be located in the PM. After 60 min the FM4-64 was located in numerous discrete punctae many of which co-localized with AtPAT10-GFP (Fig. 10b).

We next carried out co-localization studies of AtPAT10-YFP and a range of cell membrane compartment markers in crosses with Wave-mCherry lines (Geldner *et al*., 2009). Examination of the progeny of these crosses revealed significant co-localization of many of the fluorescent punctae of AtPAT10-YFP fluorescence with the Golgi stack marker Got1 (Wave 18R), and the Golgi markers SYP32 (Wave 22R) and MEMB12 (Wave 127R) (Figs 10c, S7). A few fluorescent punctae of AtPAT10-YFP were also found to co-localize with the trans-Golgi network/early endosome (TGN/EE) marker VTI12 (Wave 13R) (Fig. 10d). Therefore, AtPAT10 appears to be localized to the tonoplast and Golgi and to other discrete punctae that might be endomembrane compartments such as early endosomes. Time-lapse imaging over a period of 5 min showed that these punctae moved extensively in root cells along cytoplasmic strands, and were particularly concentrated at the basal and apical ends of root cortical cells (Movies S2–S4).

## Discussion

We have demonstrated that the Arabidopsis gene At3g51390 is required for the normal growth and development of Arabidopsis and encodes an *S*-acyltransferase, AtPAT10, that can complement the yeast *S*-acyltransferase AKR1 loss-of-function mutant *akr1* (Fig. 1), and that it is auto-acylated (Fig. 2). Furthermore, we proved that both activities of AtPAT10 require the Cys in the conserved DHHC motif because mutation of this residue to Ala prevents complementation and auto-acylation in yeast. In addition, the loss-of-function of AtPAT10 mutant *atpat10* phenotypes were rescued by ectopic over-expression of the AtPAT10 cDNA (Fig. 8), proving that the mutant phenotype is caused by the AtPAT10 gene being rendered nonfunctional. However, the AtPAT10C^192^A construct which carries a point mutation in the *S*-acyltransferase active site failed to rescue the mutant phenotype, confirming that the phenotype is caused by loss of AtPAT10 *S*-acyltransferase activity. Taken together, our data demonstrated that AtPAT10 is an *S*-acyltransferase, its enzyme activity requires the core DHHC motif, and that it is functionally independent of the other 23 Arabidopsis PATs.

Of the 24 DHHC-CRD containing proteins encoded by the Arabidopsis genome, only one, TIP1, has been functionally characterized (Hemsley *et al*., 2005). The fact that mutation of the DHHC motif of TIP1 to DHHA abolished its ability to rescue the yeast *akr1* mutant, as well as its ability to auto-acylate in yeast (Hemsley *et al*., 2005), suggests that in Arabidopsis the Cys of the DHHC motif of this family of proteins may be necessary for catalytic activity, as it is known to be in yeast and mammals (Roth *et al*., 2002; Hou *et al*., 2009; Fukata & Fukata, 2010; Mitchell *et al*., 2010). Nevertheless, the phenotypes of the T-DNA insertion mutants in these two Arabidopsis PATs are distinct, suggesting that they function in different processes.

In order to understand where AtPAT10 is expressed within cells we transformed *atpat10-1* mutant plants using *35S:AtPAT10-GFP* and *35S:AtPAT10-YFP*. The fact that both constructs fully rescued the mutant phenotype of *atpat10-1* strongly suggests that some, if not all, of the expression we observed reflects the localization of endogenous AtPAT10. In combination with FM4-64 labelling and marker WAVE lines (Geldner *et al*., 2009), we demonstrate that the AtPAT10-YFP protein was predominately located in the Golgi and tonoplast in Arabidopsis leaf, root and hypocotyl cells (Figs 9, 10). FM4-64 labels a variety of cellular membrane compartments in a variety of plant cells among which are Golgi (Bolte *et al*., 2004), trans-Golgi network/early endosome (TGN/EE) (Geldner *et al*., 2003; Dettmer *et al*., 2006; Viotti *et al*., 2010), multivesicular bodies (MVB) (Otegui & Spitzer, 2008) and tonoplast (Bolte *et al*., 2004; Tse *et al*., 2004; Geldner *et al*., 2009). Our data obtained from crosses with marker Wave lines reveal that AtPAT10 co-localizes with the three Golgi markers, Got1p (18R), SYP32 (R22) and MEMB12 (R127) (Conchon *et al*., 1999; Rancour *et al*., 2002; Uemura *et al*., 2004; Chatre *et al*., 2005; Geldner *et al*., 2009). Got1P has been localized in Golgi stacks by immunogold electron microscopy (Geldner *et al*., 2009). Some co-localization was also found with VTI12 (R13) (Geldner *et al*., 2009), a tans-Golgi network/early endosome marker (Sanderfoot *et al*., 2001; Uemura *et al*., 2004). The TGN is a highly mobile organelle in plants that frequently displays independent movement and only transiently associates with the Golgi stacks (Batistic *et al*., 2010; Viotti *et al*., 2010). Therefore, our combined data from FM4-64 staining and marker WAVE lines show that AtPAT10 is located in multiple highly mobile membrane compartments that include the Golgi stack, TGN/EE and tonoplast.

In agreement with our observations, the tonoplast location of AtPAT10 has previously been reported in a proteomic study of vacuoles from Arabidopsis cell culture (Jaquinod *et al*., 2007). Transient expression of GFP tagged AtPAT10 driven by the Mannopine Synthase gene promoter in tobacco leaf epidermal peels demonstrated localization in both tonoplast and Golgi (Batistič, 2012). Interestingly, however, a recent study using stably transformed Arabidopsis expressing C-terminal GFP tagged AtPAT10 only showed a tonoplast localization for the protein in roots; localization in Golgi or other subcellular compartments was not observed (Zhou *et al*., 2013). In the study by Zhou *et al*., the AtPAT10 C-terminal GFP fusion was driven by a putative endogenous promoter region of *c*. 1.1 kb. However, using the same promoter to make a GUS fusion the authors were unable to detect GUS signal in most reproductive tissues where *AtPAT10* is highly expressed and severe morphological defects are observed in mutant lines (Fig. 4 and Zhou *et al*., 2013). Thus, the lack of Golgi localization reported by Zhou *et al*. could be explained by the use of incomplete promoter regions in their constructs which could lead to suboptimal levels of PAT10 expression. Our observations add further detail to the cellular location of AtPAT10 in the AtPAT10 loss-of-function mutant of Arabidopsis rescued by stable transformation with AtPAT10-GFP and YFP constructs.

Of the 24 Arabidopsis PATs transiently overexpressed in tobacco epidermal peels, seven have been reported to be located in the Golgi and others are thought to reside in vesicles that co-localize with the plant endosomal marker AtRAB5C/RABF1/ARA6 (Ueda *et al*., 2001; Batistič, 2012). A dual location of ER/Golgi or Golgi/PM has been reported for several mammalian PATs and this is thought to be important for continuous cycling of proteins between membrane compartments and correct membrane localized functioning (Ohno *et al*., 2006; Greaves & Chamberlain, 2007). The location of AtPAT10 in multiple membrane compartments has not been reported for other PATs. This feature might be unique to AtPAT10, but confirming this will require detailed studies of the location of other AtPATs in stably transformed Arabidopsis.

Recent evidence suggests that the Golgi is the site of the core *S*-acylation machinery for palmitoylation of peripheral proteins in mammalian cells where acylation of proteins directs them to the secretory pathway and plasma membrane. From here they become redistributed to other cellular membranes and are ultimately de-acylated. Because most of these proteins have other lipid modifications such as myristoylation or prenylation, they are re-directed back to the Golgi where they can again be acylated and re-enter the secretory pathway (Rocks *et al*., 2010). Localization of AtPAT10 in the Golgi of Arabidopsis may reflect a similar mechanism in higher plants. However, Batistič (2012) has suggested that the plant cellular *S*-acylation machinery is functionally different compared with that of yeast and mammals because half of the AtPATs were seen to be localized to the PM in tobacco leaf peels. The yeast *S*-acyl transferase PFA3 is a vacuolar-localized PAT that palmitoylates the vacuolar fusion factor Vac8 and promotes vacuolar fusion (Hou *et al*., 2005; Smotrys *et al*., 2005). The fact that we observe AtPAT10-YFP in the tonoplast of pro-vacuoles and the mature vacuole may indicate a similar function for this Arabidopsis PAT.

Loss of *AtPAT10* function affects vascular development (Figs 7, S5). In the Arabidopsis shoot, xylem and phloem are specified from procambial cells by a complex transcriptional network comprising two types of transcription factors, HD-ZIP IIIs, and KANADIs (KANs) and the microRNAs 165/166 which are regulated by auxin and BR signalling. Mutations in many of these transcription factors affect vascular development (Caño-Delgado *et al*., 2010). For example, loss-of-function of one component of this transcriptional network, *Ifll/Rev*, causes a complete absence of interfascicular fibres (Zhong & Ye, 2001), raising the possibility that AtPAT10 may have a function in the xylary fibre developmental pathway that includes Ifl1.

The reduction in number of lignified xylem and interfascicular fibre cells in *atpat10-1* suggests a possible link with lignin biosynthesis. Cinnamyl alcohol dehydrogenase-C and -D (CAD-C, CAD-D), and cinnamoyl CoA reductase (CCR) are the primary genes involved in lignin biosynthesis in the interfascicular fibre and xylem of the Arabidopsis floral stem (Sibout *et al*., 2005). A triple *cad c cad d ccr1* mutant, *ccc*, has a significantly reduced level of lignin in mature stems and displays a severe dwarf phenotype and male sterility (Thévenin *et al*., 2011). This raises a possibility that AtPAT10 might have some role in lignin biosynthesis.

Proteins having diverse functions in plants are known to be palmitoylated, for example, the heterotrimeric G protein alpha subunit GPA1 and the gamma subunit 2 AGG2 (Adjobo-Hermans *et al*., 2006; Zeng *et al*., 2007). While the AGG2 knockout has no obvious growth defects (Trusov *et al*., 2008), null mutants of GPA1, *gpa1-4* for example, exhibit slightly rounded leaves, reduced cell division and hypersensitivity to ABA, PAC and glucose in seed germination and early seedling development (Ullah *et al*., 2001; Chen *et al*., 2006). Some small G proteins, such as Type I and II ROPs (Rho of Plants) and AtRABF1, are also shown to be *S*-acylated (Lavy *et al*., 2002; Grebe *et al*., 2003; Lavy & Yalovsky, 2006; Sorek *et al*., 2010). The *S*-acylated Calcineurin B-like (CBL) proteins in Arabidopsis are involved in K^+^ transport, salt tolerance and ABA signalling (Cheong *et al*., 2003; Pandey *et al*., 2004; Li *et al*., 2006). Recently the tonoplast localized CBL2, 3 and 6 proteins have been shown to be mislocalized in transiently transformed protoplasts prepared from *atpat10* cells. This could suggest that AtPAT10 is the palmitoylating enzyme for CBL2/3/6, although a direct protein–protein interaction between AtPAT10 and CBL2/3/6 could not be demonstrated due to technical difficulties (Zhou *et al*., 2013). However, the knockout out mutants of these CBLs individually did not exhibit an obvious phenotype (Batistic *et al*., 2010; Batistič *et al*., 2012) although double mutants of *cbl2 cbl3* did share some phenotypic defects found in *pat10* (Tang *et al*., 2012).

While the above *S*-acylated proteins are involved in various aspects of plant growth and development, none of them, however, displayed an identical phenotype to the *atpat10* mutants. Therefore, the *atpat10* phenotype may reflect the failure in *S*-acylation of more than one of these proteins, or of an as yet unidentified *S*-acylated protein or proteins.

Our identification and characterization of AtPAT10 defines a Golgi and tonoplast located Arabidopsis PAT that is functionally independent of other members of this multigene family, and demonstrates a growing importance of protein *S*-acylation in plants.
