# Supplementary material for: A Golgi and tonoplast localized S-acyl transferase is involved in cell expansion, cell division, vascular patterning and fertility in Arabidopsis
Source: New Phytol. 2013 Jun 25;200(2):444–56. doi: 10.1111/nph.12385 (PMC3817529; doi:10.1111/nph.12385)
Supplement: Supplementary file 1 [file nph0200-0444-SD1.pptx]

## Slide 1
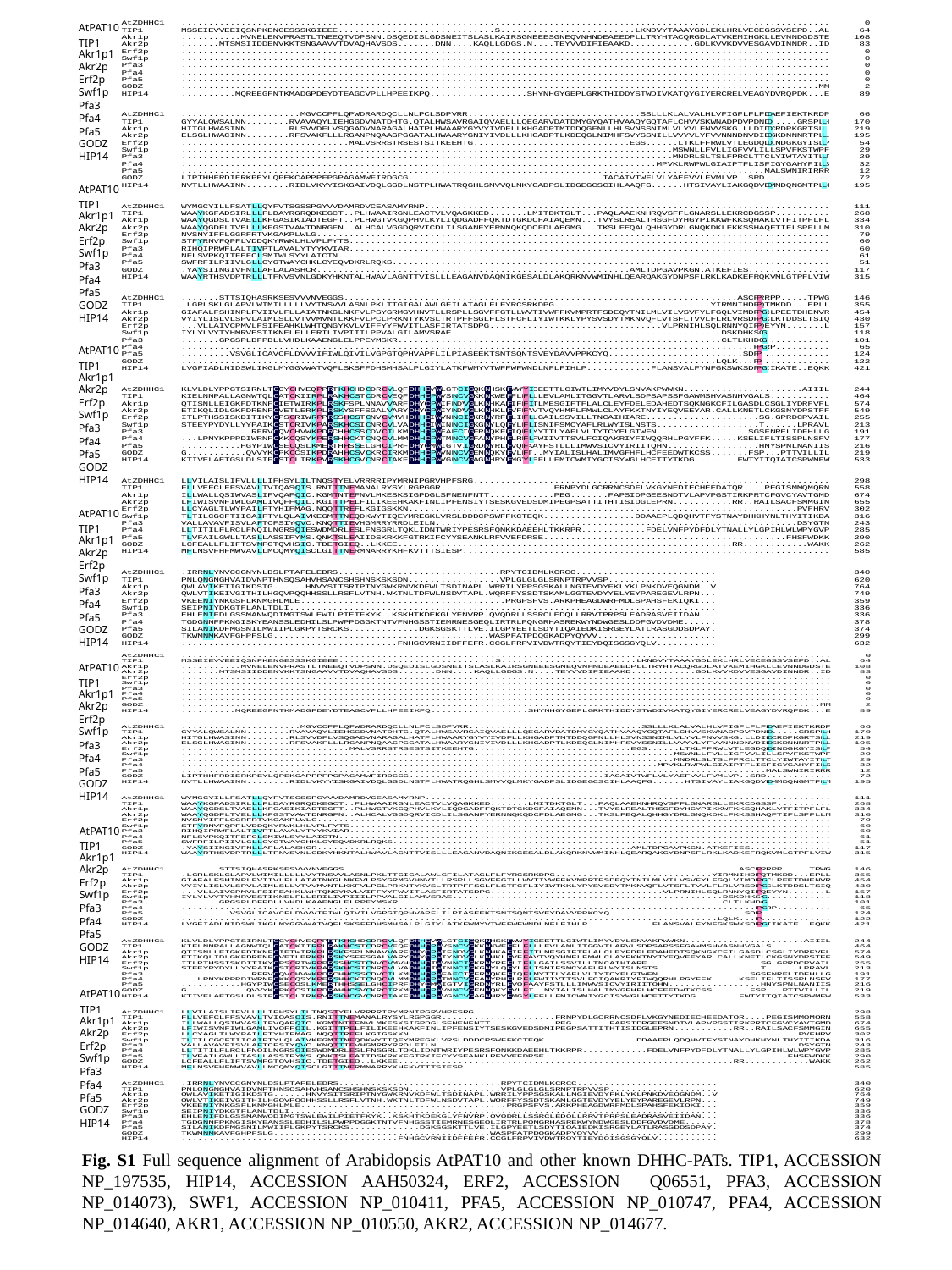

AtPAT10
TIP1
Akr1p1
Akr2p
Erf2p
Swf1p
Pfa3
Pfa4
Pfa5
GODZ
HIP14
AtPAT10
TIP1
Akr1p1
Akr2p
Erf2p
Swf1p
Pfa3
Pfa4
Pfa5
GODZ
HIP14
AtPAT10
TIP1
Akr1p1
Akr2p
Erf2p
Swf1p
Pfa3
Pfa4
Pfa5
GODZ
HIP14
AtPAT10
TIP1
Akr1p1
Akr2p
Erf2p
Swf1p
Pfa3
Pfa4
Pfa5
GODZ
HIP14
AtPAT10
TIP1
Akr1p1
Akr2p
Erf2p
Swf1p
Pfa3
Pfa4
Pfa5
GODZ
HIP14
AtPAT10
TIP1
Akr1p1
Akr2p
Erf2p
Swf1p
Pfa3
Pfa4
Pfa5
GODZ
HIP14
AtPAT10
TIP1
Akr1p1
Akr2p
Erf2p
Swf1p
Pfa3
Pfa4
Pfa5
GODZ
HIP14
Fig. S1 Full sequence alignment of Arabidopsis AtPAT10 and other known DHHC-PATs. TIP1, ACCESSION NP_197535, HIP14, ACCESSION AAH50324, ERF2, ACCESSION Q06551, PFA3, ACCESSION NP_014073), SWF1, ACCESSION NP_010411, PFA5, ACCESSION NP_010747, PFA4, ACCESSION NP_014640, AKR1, ACCESSION NP_010550, AKR2, ACCESSION NP_014677.

## Slide 2
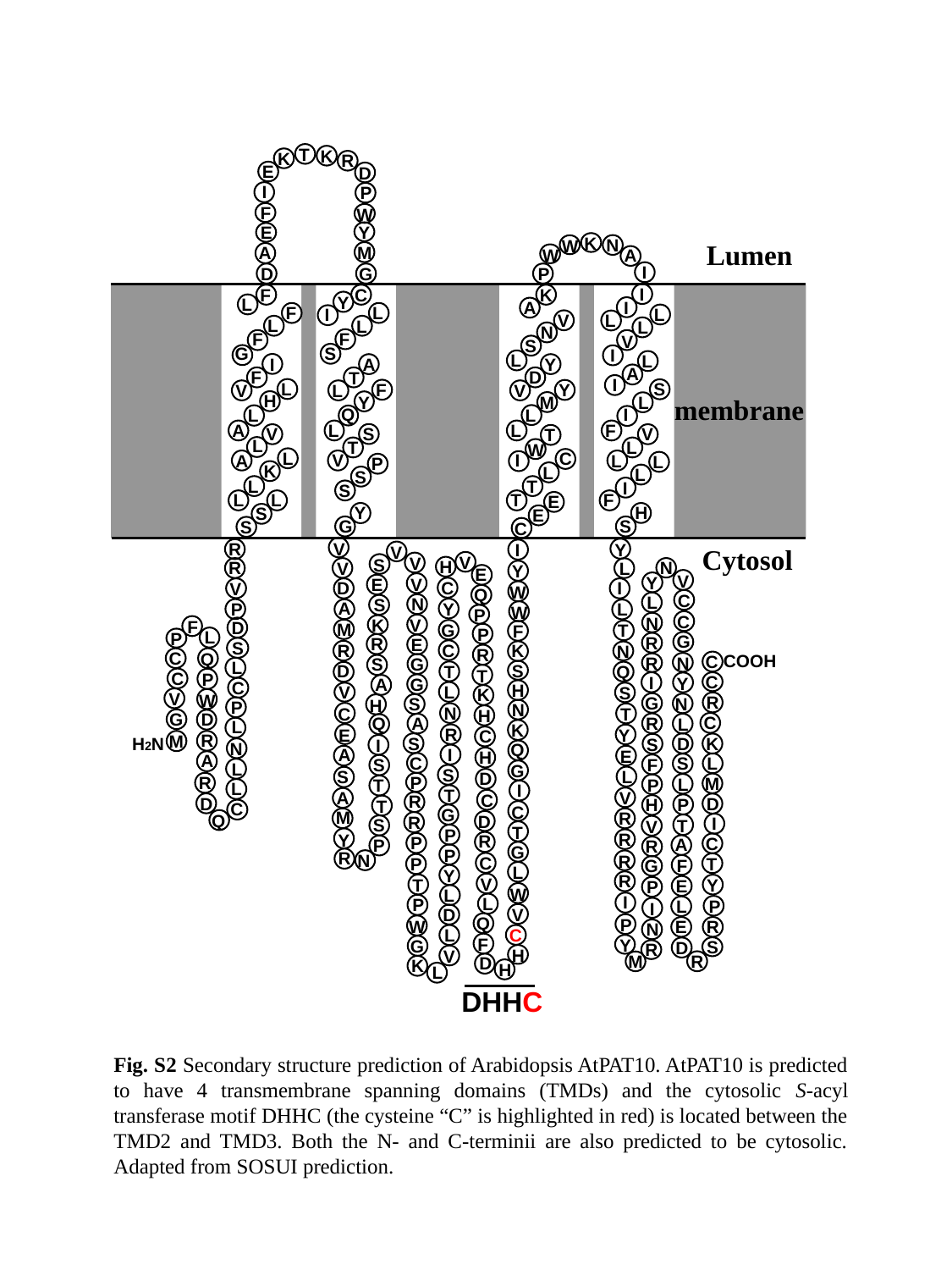

T
K
K
R
E
D
I
P
F
W
E
Y
K
N
W
Lumen
M
A
W
A
I
G
P
D
I
I
L
F
L
C
Y
I
K
A
F
L
F
G
L
L
F
S
L
L
V
I
V
N
S
L
L
A
I
I
F
V
A
Y
D
V
T
L
H
L
A
S
L
F
I
F
Y
Q
L
Y
M
L
L
L
membrane
S
V
T
V
A
V
L
T
W
I
L
L
L
C
T
L
T
L
L
F
I
P
S
S
K
L
L
L
E
Y
H
S
E
G
S
S
C
R
V
I
Y
V
Cytosol
V
V
S
H
N
R
L
V
Y
E
V
Y
E
V
C
I
D
V
W
Q
C
L
N
S
A
P
L
Y
W
P
C
N
K
V
F
D
M
G
T
F
P
L
P
G
R
R
E
S
R
C
K
N
R
C
Q
P
W
D
R
A
COOH
C
R
N
G
S
L
S
D
Q
T
T
C
C
I
Y
G
A
C
H
L
V
S
K
V
R
G
S
N
C
L
K
D
L
S
M
L
D
P
I
T
C
A
T
F
H
P
N
N
C
T
H
G
R
Q
A
L
K
R
E
Y
C
M
H2N
S
S
I
N
Q
A
I
E
H
C
S
F
L
G
S
S
L
D
R
P
T
P
L
I
T
A
V
C
R
D
H
T
C
C
G
M
R
Q
D
R
S
V
T
P
R
Y
R
P
P
R
G
P
R
N
R
C
P
G
L
Y
R
V
T
Y
E
P
W
L
I
L
P
P
L
I
D
V
Q
P
W
E
R
N
L
C
F
Y
G
S
D
R
H
V
R
M
D
K
H
L
DHHC
Fig. S2 Secondary structure prediction of Arabidopsis AtPAT10. AtPAT10 is predicted to have 4 transmembrane spanning domains (TMDs) and the cytosolic S-acyl transferase motif DHHC (the cysteine “C” is highlighted in red) is located between the TMD2 and TMD3. Both the N- and C-terminii are also predicted to be cytosolic. Adapted from SOSUI prediction.

## Slide 3
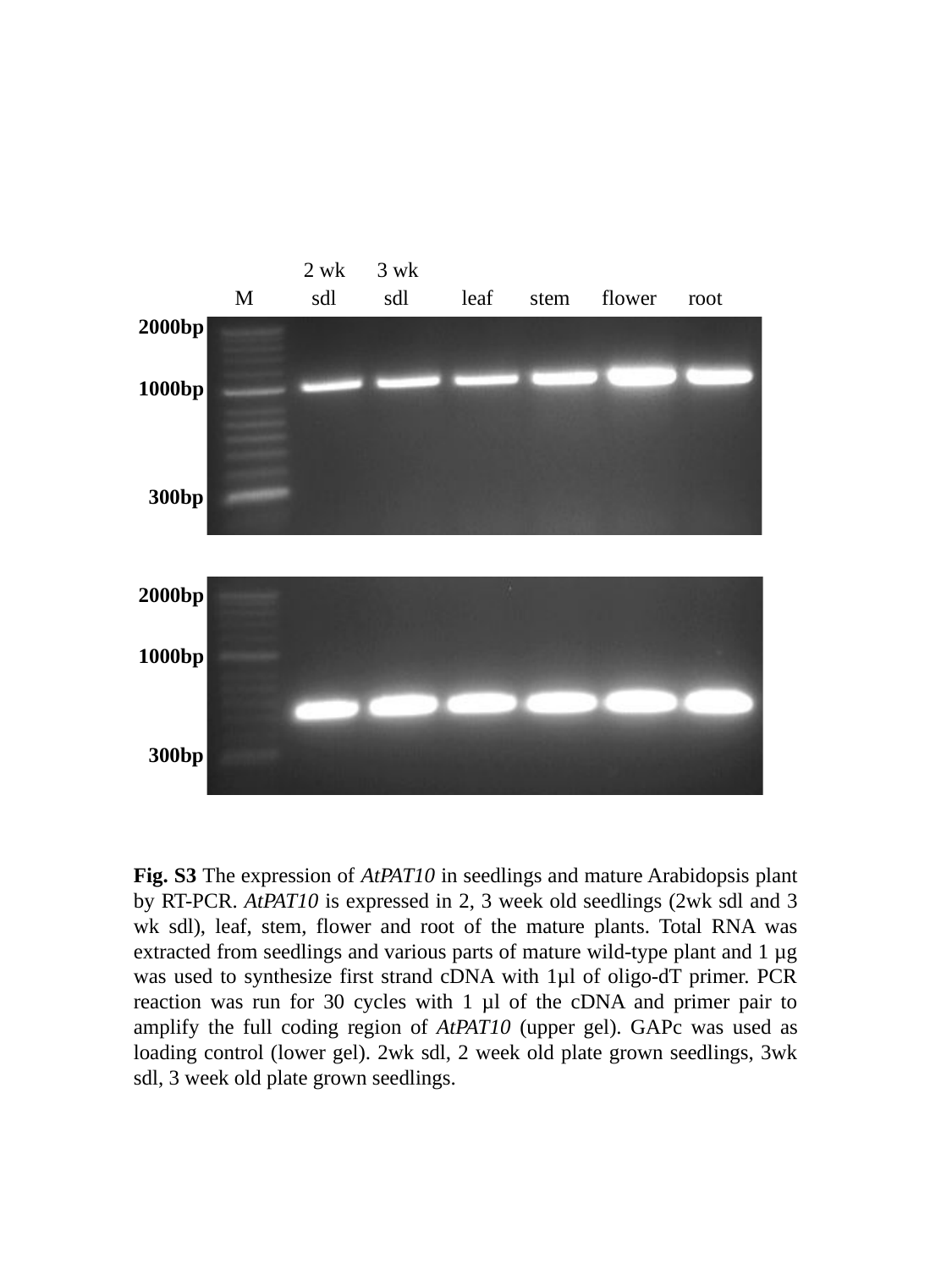

2 wk 3 wk
 M sdl sdl leaf stem flower root
2000bp
1000bp
300bp
2000bp
1000bp
300bp
Fig. S3 The expression of AtPAT10 in seedlings and mature Arabidopsis plant by RT-PCR. AtPAT10 is expressed in 2, 3 week old seedlings (2wk sdl and 3 wk sdl), leaf, stem, flower and root of the mature plants. Total RNA was extracted from seedlings and various parts of mature wild-type plant and 1 µg was used to synthesize first strand cDNA with 1µl of oligo-dT primer. PCR reaction was run for 30 cycles with 1 µl of the cDNA and primer pair to amplify the full coding region of AtPAT10 (upper gel). GAPc was used as loading control (lower gel). 2wk sdl, 2 week old plate grown seedlings, 3wk sdl, 3 week old plate grown seedlings.

## Slide 4
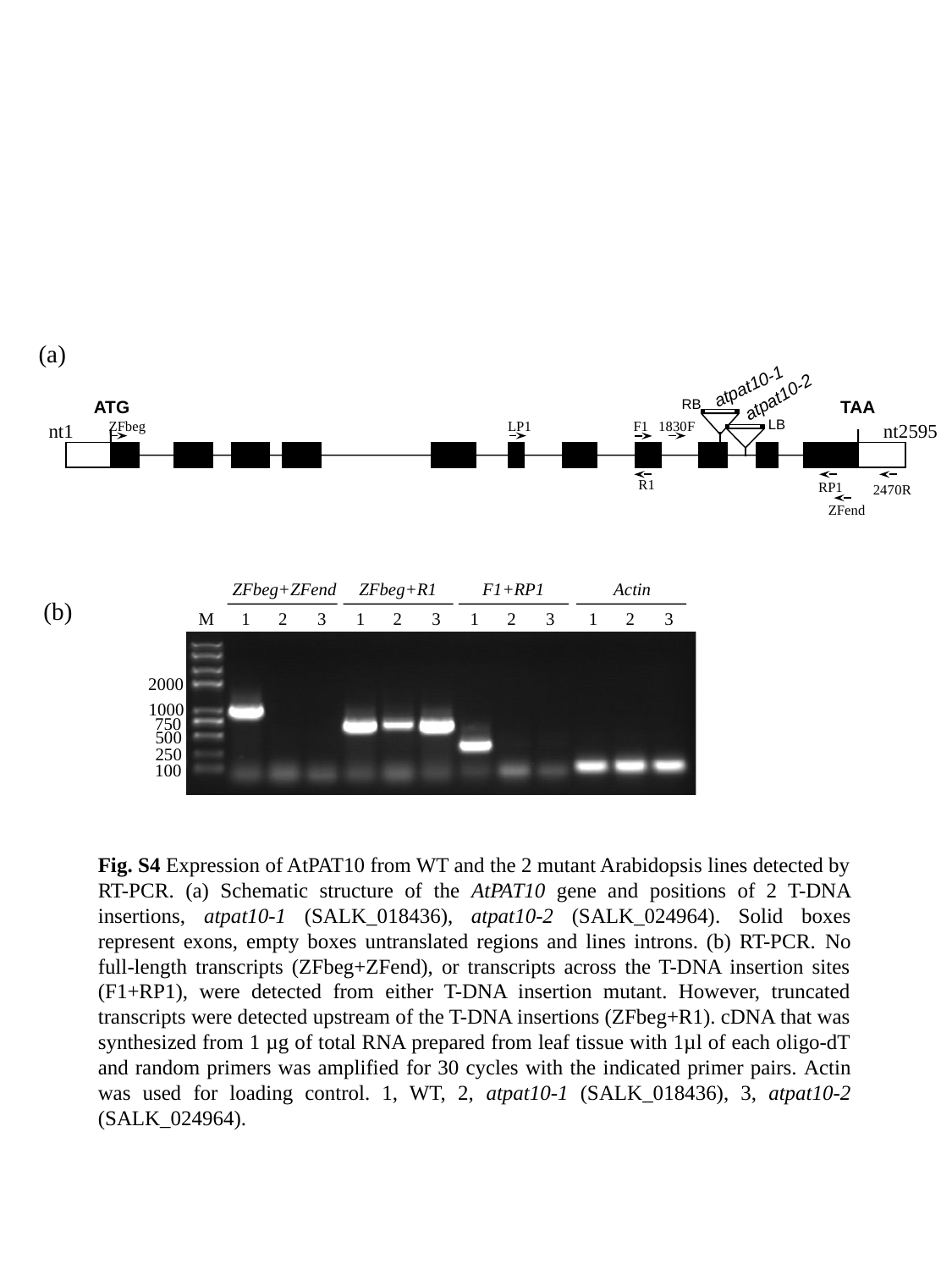

(a)
atpat10-1
atpat10-2
ATG
TAA
RB
LB
ZFbeg
LP1
F1
1830F
nt1
nt2595
R1
RP1
2470R
ZFend
ZFbeg+ZFend
ZFbeg+R1
F1+RP1
Actin
M
1
2
3
1
2
3
1
2
3
1
2
3
2000
1000
 750
 500
 250
 100
(b)
Fig. S4 Expression of AtPAT10 from WT and the 2 mutant Arabidopsis lines detected by RT-PCR. (a) Schematic structure of the AtPAT10 gene and positions of 2 T-DNA insertions, atpat10-1 (SALK_018436), atpat10-2 (SALK_024964). Solid boxes represent exons, empty boxes untranslated regions and lines introns. (b) RT-PCR. No full-length transcripts (ZFbeg+ZFend), or transcripts across the T-DNA insertion sites (F1+RP1), were detected from either T-DNA insertion mutant. However, truncated transcripts were detected upstream of the T-DNA insertions (ZFbeg+R1). cDNA that was synthesized from 1 µg of total RNA prepared from leaf tissue with 1µl of each oligo-dT and random primers was amplified for 30 cycles with the indicated primer pairs. Actin was used for loading control. 1, WT, 2, atpat10-1 (SALK_018436), 3, atpat10-2 (SALK_024964).

## Slide 5
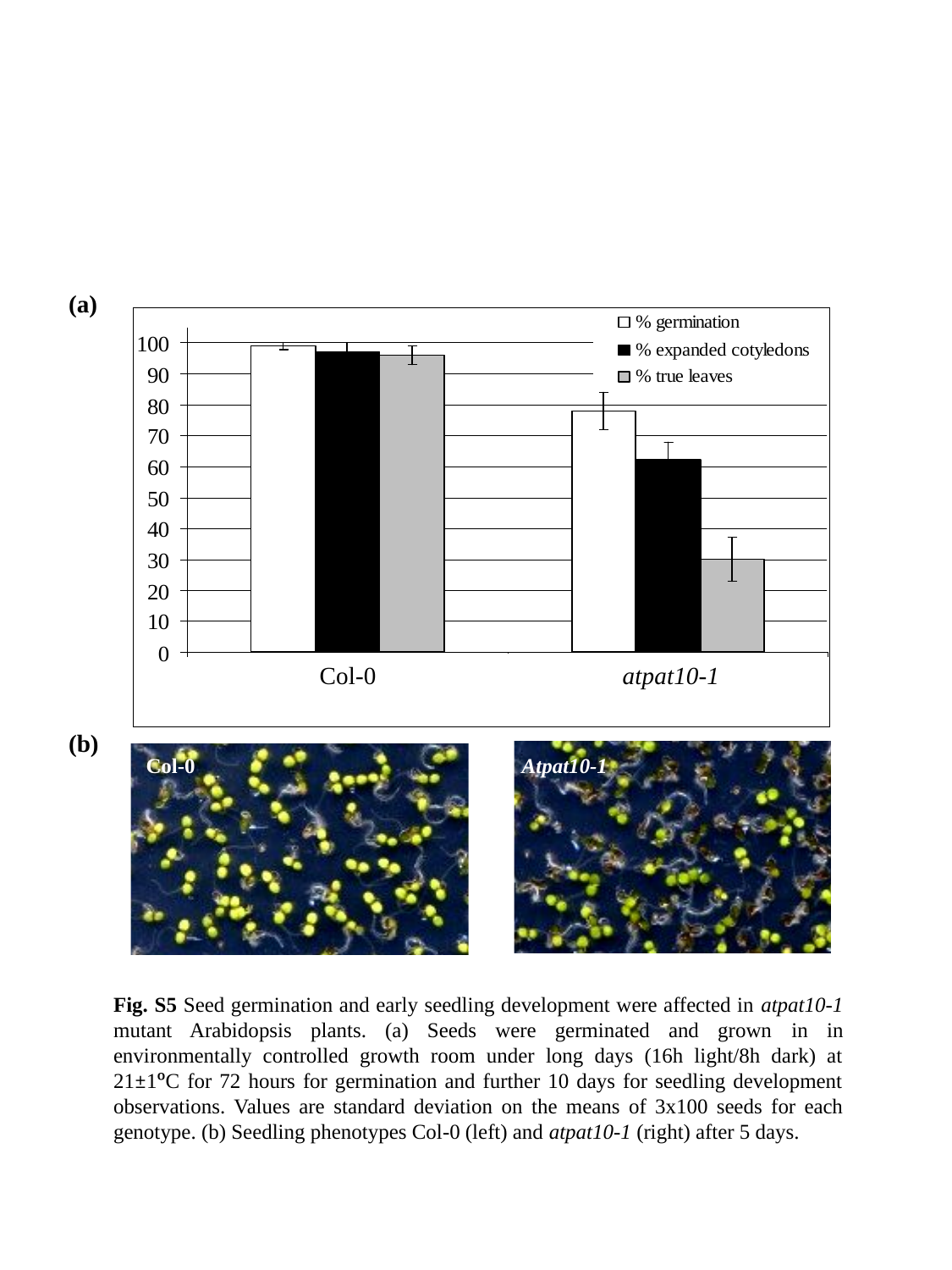

(a)
Col-0 		 atpat10-1
(b)
Col-0
Atpat10-1
Fig. S5 Seed germination and early seedling development were affected in atpat10-1 mutant Arabidopsis plants. (a) Seeds were germinated and grown in in environmentally controlled growth room under long days (16h light/8h dark) at 21±1ºC for 72 hours for germination and further 10 days for seedling development observations. Values are standard deviation on the means of 3x100 seeds for each genotype. (b) Seedling phenotypes Col-0 (left) and atpat10-1 (right) after 5 days.

## Slide 6
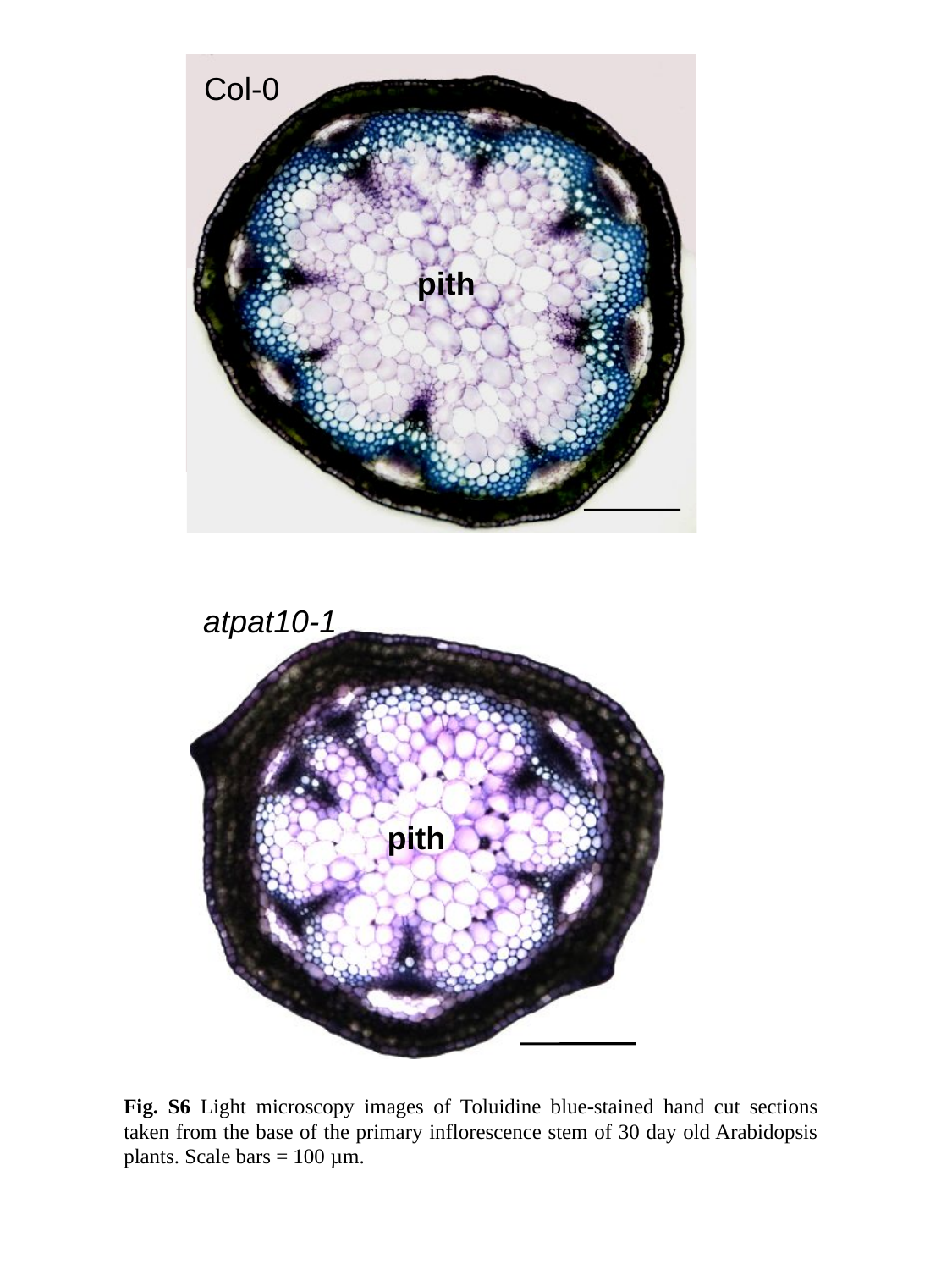

Col-0
pith
atpat10-1
pith
Fig. S6 Light microscopy images of Toluidine blue-stained hand cut sections taken from the base of the primary inflorescence stem of 30 day old Arabidopsis plants. Scale bars = 100 µm.

## Slide 7
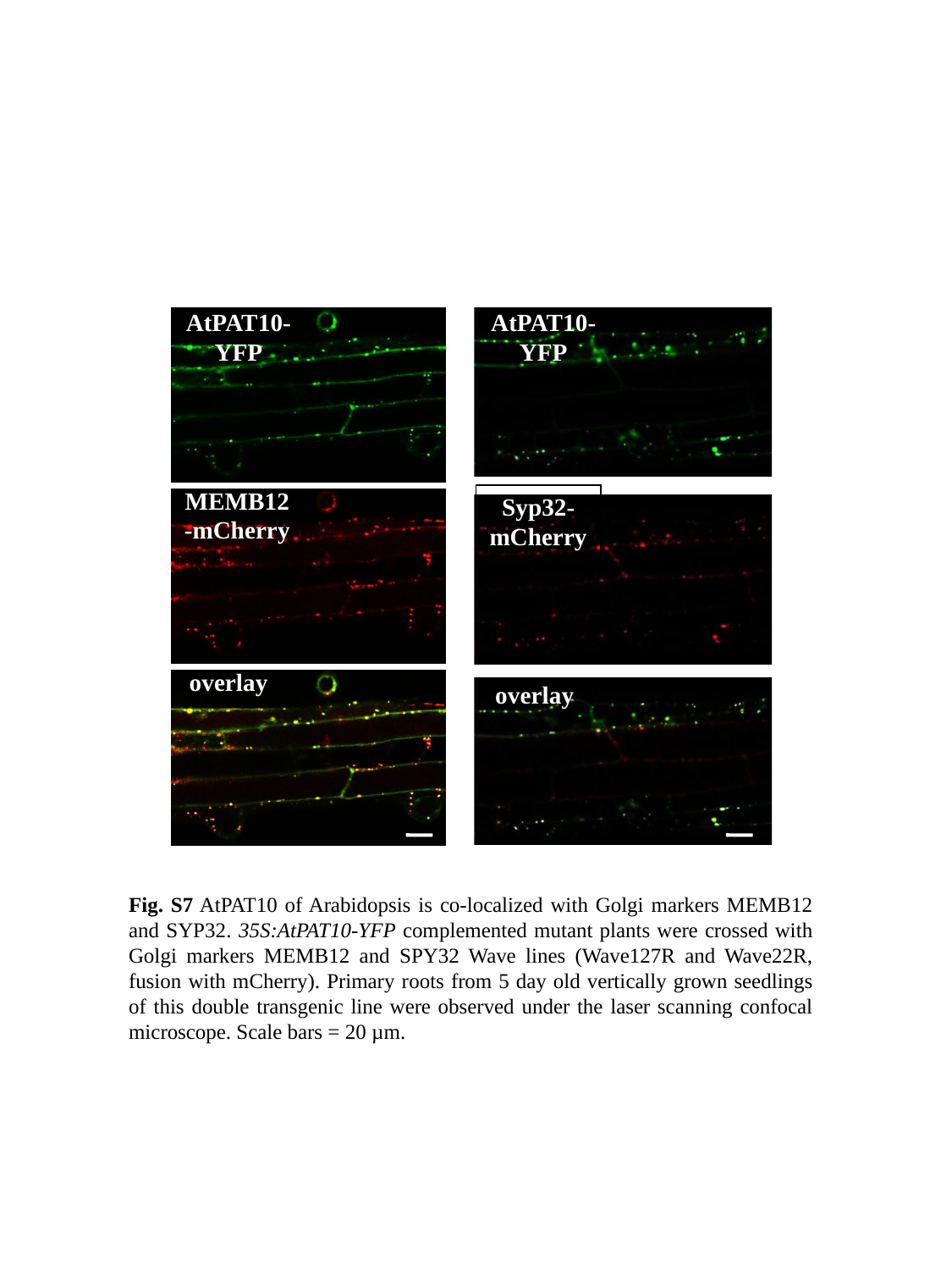

AtPAT10-YFP
AtPAT10-YFP
MEMB12-mCherry
Syp32-mCherry
overlay
overlay
Fig. S7 AtPAT10 of Arabidopsis is co-localized with Golgi markers MEMB12 and SYP32. 35S:AtPAT10-YFP complemented mutant plants were crossed with Golgi markers MEMB12 and SPY32 Wave lines (Wave127R and Wave22R, fusion with mCherry). Primary roots from 5 day old vertically grown seedlings of this double transgenic line were observed under the laser scanning confocal microscope. Scale bars = 20 µm.
