# Supplementary material for: A Golgi and tonoplast localized S-acyl transferase is involved in cell expansion, cell division, vascular patterning and fertility in Arabidopsis
Source: New Phytol. 2013 Jun 25;200(2):444–56. doi: 10.1111/nph.12385 (PMC3817529; doi:10.1111/nph.12385)
Supplement: Supplementary file 2 [file nph0200-0444-SD2.docx]

**Supporting Information Tables S1–S3**

**Table S1** Phenotypic analysis of Col-0 and *atpat10-1*

|  | Col-0 | *atpat10-1* |
| --- | --- | --- |
| Leaf Length (mm) | 12.9 ± 1.4 | 4.7 ± 0.3 |
| Leaf Width (mm) | 8.8 ± 1.0 | 3.31 ± 0.2 |
| Petiole length (mm) | 5.7 ± 1.0 | 1.6 ± 0.3 |
| Leaf Area (mm2) | 91.9 ± 20.2 | 13.0 ± 1.6 |
| Rosette diameter (mm) | 127.5 ± 6.1 | 43.0 ± 4.7 |
| Plant height (mm) | 202.4 ±16.1 | 422.8±23.3 |
| Distance from rosette to 2nd node (mm) | 44.5 ± 8.2 | 22.9 ± 3.6 |
| No. of branches | 55 ± 6 | 14 ± 2.8 |
| No. of siliques per plant | 400 ± 20 | 10.0 ± 5.4 |
| No. of ovules per pistil | 60 ± 4 | 40 ± 2 |
| No. of seeds per silique | 58 ± 3 | 5 ± 3 |
| Length of silique (mm) | 17 ± 1.4 | 4 ± 1.7 |

Leaf area, length, width and petiole length were measured from 3 wk old plants using the largest, most expanded leaf. The *atpat10-1* data sets were based on measurements from 6 leaves whilst Col-0 data was based on the measurements of 7 leaves. The distance from the base of the flowering stem to the second node (wt *n*=14, *atpat10-1* *n*=13) were measured on 39 d old plants while the plant heights and number of branches were measured on 8 wk old plants from Col-0 (*n*=8) and 12 wk from *atpat10-1* (*n*=16) plants. Number of ovules per pistil was counted from 16 fully opened flowers for both genotypes and the number of seeds per silique from 20 mature pods.

**Table S2** Cell size and number

|  | Col-0 | | *atpat10-1* | |
| --- | --- | --- | --- | --- |
|  | Size (μm^2^) | No. | Size (μm^2^) | No. |
| Parenchyma | 8926.3 ±182.0 | 114 ±7 | 5483.8 ±113.2 | 65 ±10 |
| Petal | 203.4 ±18.0 | 98.4 ±6.7 | 138.3±10.0 | 66.1±6.9 |

Cell size was estimated by measuring the area of 70 parenchyma cells in the pith of longitudinal sections of the base of the primary inflorescence stems stained with toluidine blue and 100 epidermal cells immediately above the elongated epidermal cells of the claw of the adaxial side of cleared petals from fully opened flowers of Col-0 (*n*=8) (C), and *atpat10-1* (*n*=12) (D) of 30-d-old plants. Epidermal cell number in petals was counted at the widest part of the blade. Values are means ± standard deviation.

**Table S3** Sequence of primers used

| Primer | sequence | Restriction site |
| --- | --- | --- |
| DHHCtoA F | 5’-GTTTGATCATCAC***gc***TGTTTGGTTAGGAAC-3’ |  |
| DHHCtoA R | 5’-GTTCCTAACCAAACA***gc***GTGATGATCAAAC-3’ |  |
| ZFendE | 5’-GAATTCGTGCAGCAGCGACATTTCAAC-3’ | *EcoR*I |
| ZFbegK | 5’-GGTACCGAACC**A**T**G**GGCGTTTGTTGCCCTTTCC-3’ | *Kpn*I |
| LBb1.3 | 5’-ATTTTGCCGATTTCGGAAC-3’ |  |
| RBa1 | 5’-GTTTCTGACGTATGTGCTTAGC-3’ |  |
| zfRP1 | 5’-TATCGATGCAGGTGTAGGGTC-3’ |  |
| zfLP | 5’-CAGAAATTTGACATGCGGCTATTGTC-3’ |  |
| ZFbeg | 5’-ATGGGCGTTTGTTGCCCTTTC-3’ |  |
| ZFend | 5’-TTAGCAGCAGGACATTTCAAC-3’ |  |
| ZFR1 | 5’-GTCAACGTACATGATGAGCG-3’ |  |
| actinF | 5’-AACGACCTTAATCTTCATGGTCC-3’ |  |
| actinR | 5’-GGTAACATTGTGCTCAGTGGTGG-3’ |  |
| 1830F | 5’-ACTTGTTAAAACACGAATTTC-3’ |  |
| 2470R | 5’- GGAATCCTTGTATGTATACAATATCATC-3’ |  |

The changed nucleotide bases in mutagenesis primers DHHCtoA F and DHHCtoA R are indicated by bold italic type and underlined. Restriction sites in other primers are underlined.
