# Supplementary material for: A Golgi and tonoplast localized S-acyl transferase is involved in cell expansion, cell division, vascular patterning and fertility in Arabidopsis
Source: New Phytol. 2013 Jun 25;200(2):444–56. doi: 10.1111/nph.12385 (PMC3817529; doi:10.1111/nph.12385)
Supplement: Supplementary file 3 [file nph0200-0444-SD3.doc]

**Supporting Information Methods S1**

**Methods S1** Additional materials and methods information

**Growth and transformation of Arabidopsis**

Arabidopsis seeds were surfaced sterilized, sown on 0.8% (w/v) phytogel (Sigma) with 0.5x MS basal salts (0.5xMS) (Melford), stratified and transferred to an environmental controlled growth chamber maintained at 21±1°C, 60% relative humidity and 16 h light, 120 μm m^-2^ s^-1^ / 8 h dark. After 7-10 d they were potted in Levingston F2 and grown to maturity under the same conditions. Transgenics were obtained by floral dipping (Clough & Bent, 1998) and selected on 10 mg l^-1^ glufosinate ammonium (Qi *et al*., 2004).

**Acyl-biotinyl exchange (ABE) assay**

10ml of yeast cells (wild-type strain YPH500, Stratagene) containing the pYES-AtPAT10 and pYES-AtPAT10C^192^A (C-terminal fusion with V5 epitope tag located on the vector) were grown in minus uracil yeast minimal medium containing 2% galactose at 25^o^C with shaking overnight. Cells were harvested and resuspended in 500 µl lysis buffer containing 1xPBS (pH 7.4), protein inhibitor cocktail (Roche), 1 mM EDTA, 1% Triton X-100 and 25 mM N-ethylmaleimide (NME). Half grams of acid washed sands (50–200 μM, Sigma) were added to the cells and the tubes were vortexed 10 s followed by cooling down on ice for 1 min. This procedure was repeated 6 times or until most of the cells were broken open. The samples were incubated at 4 °C for 1 h on a rotator and centrifuged at 500 ***g*** for 10 min at 4°C to remove insoluble materials. The total protein content in the supernatant was estimated by the Bradford assay (Sigma). One milligram of the total protein were combined with lysis buffer to a total volume of 1 ml and this was incubated overnight at 4°C whilst rotating to further block free –SH sites on the proteins by NEM. Proteins were precipitated at room temperature by adding 1:3:1:4 protein:methanol:chloroform:water. After vortexing the samples were centrifuge at approximately 4500 ***g*** for 2 min at 14°C. The upper phase was removed and 4 volumes of methanol were added, mixed well and centrifuged as before. The pellet was air-dried for 10 min at room temperature and resuspended in 200 μl of resuspension buffer (1xPBS, pH7.4, 8M urea and 2% SDS) by incubating at 37°C for 10–30 min with slight rocking. To hydrolyse the thiol ester bonds, the protein solution was divided into 2 x 100 µl and one was combined with 250 µl 4M fresh hydroxylamine (+NH_2_OH, pH 7.4, Sigma), 2 µl 0.5M EDTA (final conc 1 mM), 40 µl of 25x protease inhibitor (Roche) and 50 µl of 8 mM biotin-HPDP (Thermo Fisher, made fresh), bring the total volume to 1 ml by adding 558µl H_2_O. This will be the plus NH_2_OH (+) *S*-acylation sample. To the second 100 μl of proteins 808 µl 50 mM Tris pH 7.4, 2 µl 0.5M EDTA (final conc 1 mM), 40 µl of 25x protease inhibitor and 50 µl of 8 mM biotin-HPDP (made fresh). This will be the minus NH_2_OH (-) control. Both samples were gently mixed for 1 h at RT on a rotator and the proteins were precipitated as before and resuspended in 100 µl of resuspension buffer. 10 µl was removed to act as a loading control by mixing with 10 µl 2 x SDS-PAGE sample buffer. To the remaining 90 µl add 900 µl PBS containing 0.2% Triton X-100. To immobilise –SH proteins add 50 µl High Capacity Streptavidin Agarose (Thermo Fisher) to each of the + and - sample and rotate at room temperature for 1 h to capture the biotinylated proteins. Beads were collected by centrifugation followed by washing 4–5 times with 1 ml of PBS (pH 7.4) in each wash. Finally, the proteins were eluted by boiling the bead in 30 µl of 2 × SDS sample buffer for 5 min. 10 µl was loaded and resolved on a 12% SDS-PAGE gel. After transferring to the membrane, AtPAT10 and AtPAT10C^192^A were probed with anti-V5 primary antibody (rabbit polycolonal antibody, Bethyl) and detected with the secondary alkaline phosphatase-conjugated anti-rabbit antibody and NBT/BCIP® solution (both from Sigma).

**Identification of T-DNA insertion lines by PCR-genotyping**

The junctions between the ends of the T-DNA and flanking genomic DNA of AtPAT10 were amplified from homozygous plants, using T-DNA left (LBb1.3) and right border specific primers (RBa1) and the corresponding *AtPAT10* gene specific primers (zfRP1 and zfLP1) (Table S3). These products were subsequently sequenced to confirm where exactly the T-DNAs are inserted.

Mutants were back crossed to Col-0 and selfed for three generations. Homozygous lines were identified as described above and used for subsequent studies.

**RT-PCR**

RT-PCR was carried out on homozygous plants to measure AtPAT10 transcript levels. Total RNA was isolated from rosette leaves using Tripure reagent (Roche) and the first strand cDNA was reverse transcribed from 1 μg of total RNA, using the Cloned AMV First-Strand cDNA Synthesis Kit with an oligo dT plus random primers (Invitrogen). PCR reaction was run for 30 cycles with 1 µl of the cDNAs as templates. Primers ZFbegK and ZFendE (Table S3) were used to amplify the full coding region of AtPAT10. The levels of the truncated transcript were also determined in these homozygous plants from the same cDNA templates by PCR with the primer pair F1 and RP1 (regions across the T-DNAs), ZFbeg and R1 (regions between the start codon and 87 and 110 bp upstream of the T-DNA insertion site for line *atpat10-1* (SALK_018436 and *atpat10-2* (SALK_024964, respectively). The actin transcript amplified with primers actinF and actinR (Table S3) was used as a control for amplification.

**Complementation of the *atpat10* mutants**

**A**tPAT10 cDNA was amplified from an *Arabidopsis* plasmid cDNA library constructed in the pUraM (B. Qi and R Hooley, unpublished), using primers ZFbegK and ZFendE (Table S3) and KOD high fidelity polymerase (Novagen). The product was cloned into pENTR/D or pENTR 3C Dual (Invitrogen) to generate entry clones. These were then transferred by recombination utilising Gateway cloning technology (Gateway, Invitrogen) into the plant binary destination vector pEarleyGate100, 101, 103 and 202 (Earley *et al*., 2006) which has a CaMV35S promoter-nos terminator expression cassette, generating non-tagged (pEarleyGate 100), N-terminal FLAG- (pEarleyGate 202) and C-terminal GFP or YFP tagged (pEarleyGate 103 or pEarleyGate 101) AtPAT10, respectively. The recombinant binary vector was transferred to *Agrobacterium tumefaciens* strain GV3101. Because the homozygous mutant *atpat10-1* and *atpat10-2* plants do not generate many fertile flowers, heterozygous plants were used for transformation via the floral dip method (Clough & Bent, 1998). *Basta* resistant transgenic seedlings were selected as previously described (Qi *et al*., 2004). These plants were subsequently PCR-genotyped using LBb1.3 and zfRP1 to identify plants containing the T-DNA insertions. A second pair of primers 1830F and 2470R (Table S3) that are located in the 8^th^ intron and 3’ untranslated region of AtPAT10 (because zfLP1 and zfRP1 are in exons that will amplify the 35S:AtPAT10 constructs), flanking the T-DNA insertion sites, were used to identify which of these T-DNA containing transformants were also homozygous for the T-DNA insertion.

**Multiple alignments and phylogenetic analysis**

Multiple sequence alignments were performed using DNAMAN (Lynnon BioSoft). The AtPAT10 secondary structure was predicted by SOSUI programme (<http://sosui.proteome.bio.tuat.ac.j/about-sosui.html>). All programs were used in default settings.

**References**

**Clough SJ, AF.** **1998.** Floral dip: A simplified method for Agrobacterium-mediated transformation of *Arabidopsis thaliana*. *Plant Journal* **16:** 735–743.

**Earley KW, Haag JR, Pontes O, Opper K, Juehne SK, Pikaard CS. 2006.** Gateway-compatible vectors for plant functional genomics and proteomics. *Plant Journal* **45**: 616–629.

**Qi B, Fraser T, Mugford S *et al*.** **2004**. Production of very long chain polyunsaturated omega-3 and omega-6 fatty acids in plants. *Nature Biotechnology* **22:** 739–745.
