# Supplementary material for: A Golgi and tonoplast localized S-acyl transferase is involved in cell expansion, cell division, vascular patterning and fertility in Arabidopsis
Source: New Phytol. 2013 Jun 25;200(2):444–56. doi: 10.1111/nph.12385 (PMC3817529; doi:10.1111/nph.12385)
Supplement: Supplementary file 4 [file nph0200-0444-SD4.docx]

**Supporting Information legends to Movies S1–S4**

**Movie S1** Arabidopsis AtPAT10 is localized in the tonoplasts. AtPAT10-YFP is localized in the tonoplasts as well as displays as fluorescence punctuates dispersed in the cytoplasm of hypocotyl cells of seedlings of Arabidopsis. Many small tonoplasts were seen to fuse to form larger ones (arrows). Images were collected every 3 s over a period of 6 min under the laser scanning confocal microscope (Olympus) using ×60 oil immersion lens.

**Movie S2** AtPAT10-YFP is seen moving along the cytoplasmic strands rapidly in the primary root cells of Arabidopsis seedlings. Images were collected every 3 s over a period of 5 min under the laser scanning confocal microscope (Olympus) using ×60 oil immersion lens.

**Movie S3** After 5 min staining with FM4-64 (red) AtPAT10-YFP (green) is seen moving along the cytoplasmic strands rapidly in the primary root cells of Arabidopsis seedlings. Little co-locolization was observed at this stage.

**Movie S4** After 40 min of staining with FM4-64 (red) AtPAT10-YFP (green) is seen moving along the cytoplasmic strands rapidly in the primary root cells of Arabidopsis seedlings. The two fluoresence signals were largely co-localized (yellow).
